# Associations Between Healthy and Plant-Based Dietary Patterns and Cognitive Reserve: A Cross-Sectional Analysis of the 1946 British Birth Cohort

**DOI:** 10.1016/j.cdnut.2025.107599

**Published:** 2025-11-12

**Authors:** Kelly C Cara, Tammy M Scott, Paul F Jacques, Mei Chung

**Affiliations:** 1 Division of Nutrition Epidemiology and Data Science, Gerald J. and Dorothy R. Friedman School of Nutrition Science and Policy, Tufts University, Boston, MA, United States; 2 Department of Population Science, American Cancer Society, Atlanta, GA, United States; 3 Jean Mayer USDA Human Nutrition Research Center on Aging, Boston, MA, United States

**Keywords:** cohort studies, cognition, cognitive reserve, Healthy Eating Index, diet, healthy, diet, plant-based

## Abstract

**Background:**

Cognitive reserve (CR) is the ability to compensate for brain changes, injury, and disease, and it is measured by acquired knowledge and experiences. Genes and environmental factors influence CR, but the role of diet is unclear.

**Objectives:**

This study examined associations between CR and alignment with different dietary patterns and investigated diet’s unique contribution to variability in CR.

**Methods:**

We applied cross-sectional methods to data from the National Survey of Health and Development’s 1946 British Birth Cohort. National Adult Reading Test (NART) scores at age 53 identified CR levels. Scores for the Healthy Eating Index (HEI)-2020 and Plant-Based Diet Indexes (PDIs overall, healthful [hPDI], and unhealthful [uPDI]) were calculated from diet recalls and diaries at ages 4, 36, 43, and 53. Multiple linear regression models identified associations between NART and cumulative mean dietary index scores while adjusting for key confounders. Owen value *R*^2^ decomposition determined variance explained by covariate groups.

**Results:**

Participants with complete data were analyzed (*n* = 2514, 48% male). In regression models, NART was positively associated with HEI, PDI, and hPDI but inversely associated with uPDI. Compared with the lowest quintile, those in the highest quintile for HEI, PDI, and hPDI had significantly higher NART [adjusted mean difference (95% CI) for HEI: 2.25 (1.19, 3.30); PDI: 1.17 (0.19, 2.15); hPDI: 1.42 (0.51, 2.33)], whereas the highest quintile for uPDI showed lower NART (−1.55 [−2.55, −0.54]). Associations weakened but remained significant in models additionally adjusted for childhood cognitive ability. Among dietary patterns, HEI explained the most variation in NART (HEI 2.84%, uPDI 1.51%, hPDI 1.05%, PDI 0.51%).

**Conclusions:**

CR was positively associated with healthy dietary patterns and inversely associated with unhealthful plant-based dietary patterns. Diet uniquely explained variations in CR and should be considered among influential lifestyle factors in future research. Longitudinal analyses are needed to confirm these findings.

## Introduction

Cognitive decline naturally occurs with aging, but Alzheimer’s Disease (AD) and Related Dementias (ADRD) cause changes in the brain’s structure and function that lead to more severe problems with memory, thinking, and behavior [1]. However, some people with brain disease pathology typically associated with AD/ADRD show no cognitive symptoms [2]. This individual difference in clinical expression of the disease is attributed in part to cognitive reserve, which is a property of the brain that helps cope with or compensate for brain changes and brain injury or disease [3,4]. Cognitive reserve is influenced by genes and environmental factors that affect brain size and function throughout life [5]. This primarily includes education and occupation, but health behaviors and lifestyle also influence cognitive reserve [5,6]. With no known cure for AD/ADRD and few available treatments [1], evidence for the role of cognitive reserve and potentially modifiable lifestyle risk factors, like diet, is urgently needed but currently scarce. Furthermore, the influence of diet on cognitive reserve is not yet known.

Evidence suggests healthy dietary patterns rich in plant sources of food can help improve cognition and prevent, delay onset, or slow the progression of age-related degenerative brain diseases [7]. Dietary patterns rich in antioxidants and MUFAs, and with balanced ratios of essential ω-6 and ω-3 PUFAs, are particularly well suited for mitigating the oxidative stress, inflammation, and vascular risk factors associated with cognitive impairment and age-related diseases, including AD [8]. Healthy dietary patterns high in plant sources of foods that limit or exclude animal sources of foods promote more balanced ω-6 to ω-3 ratios through lower intake of SFAs and higher intake of MUFAs and PUFAs. Furthermore, plants provide a great source of dietary antioxidants in the form of polyphenols, vitamins, and carotenoids, which are known to enhance neuron function and plasticity, stimulate blood flow to and within the brain, promote the development of new neurons, and mitigate risk of neurodegenerative diseases [[9], [10], [11]].

Research also suggests that certain aspects of cognitive ability known as fluid abilities (e.g., processing speed, working memory, and reasoning) are impacted in early-stage Alzheimer’s, whereas other aspects known as crystallized abilities (e.g., reading and writing) are initially spared [12]. Crystallized abilities are rooted in knowledge and experiences acquired over time, which include general knowledge, lexical knowledge, vocabulary, and language skills. Because these abilities are less vulnerable to Alzheimer’s, they are a good indicator of premorbid cognitive ability and, therefore, of cognitive reserve. The National Adult Reading Test (NART) measures reading ability and has proven to be stable in patients aged 20–70 y, which further confirms the resilience of this crystallized ability over time [13]. The NART is commonly used by clinicians as a measure of cognitive reserve [12], so understanding the relationship between healthy dietary patterns and NART scores could help identify whether diet may play a role in cognitive reserve.

This study aimed to investigate associations between different dietary patterns and cognitive reserve using data from the 1946 British Birth Cohort. We hypothesized that greater alignment with healthy dietary patterns would be positively associated with cognitive reserve, whereas alignment with less healthy dietary patterns would show an inverse association.

## Methods

### Study population

The Medical Research Council (MRC) National Survey of Health and Development (NSHD) is an ongoing prospective cohort study developed as a follow-up to the 1946 Maternity Survey [14]. The resulting 1946 British Birth Cohort participants were a representative sample randomly selected from all regions of the United Kingdom and from diverse socioeconomic and family circumstances. The sample included 5362 singleton infants (52% male) drawn from 16,695 total births during 1 wk in March 1946, per the British birth registries [15]. This cohort has contributed data 25 times over 75 y via interviews, medical examinations, and postal questionnaires. Cohort members or their mothers (when the cohort members were children) have given written informed consent to participate in each data collection period [15,16] and to have their data stored in accordance with the Data Protection Act [17]. Ethical approval for NSHD is provided by Research Ethics Committees in both England and Scotland [16,17], and the present study received ethical approval from the MRC Unit for Lifelong Health and Ageing at University College London to utilize NSHD data from 1946 to 2005 [18], 2006 to 2012 [19], and 2013 to 2018 [20], excluding participants who withdrew from analyses. The present study protocol was reviewed and granted exempt status by the Tufts University Social, Behavioral & Educational Research institutional review board (SBER IRB; STUDY00002289).

**FIGURE 1:**
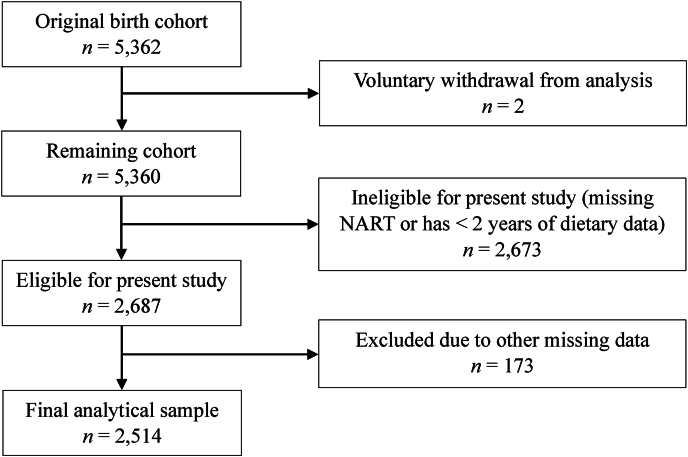
Selection process for the final analytical sample of the 1946 British Birth Cohort members.

Cohort members with NART scores at age 53 and with dietary intake data from ≥2 data collection periods up to age 53 were eligible for the present cross-sectional analysis. All participants provided data on birth sex and region. We excluded participants missing any of the following information at time points relevant to this study: household social class, education level, smoking status, exercise status, intellectual and social activities, BMI (in kg/m^2^), diabetes, heart attack, headaches/migraines, hypertension or high blood pressure, epilepsy medication use, nervous/emotional trouble or anxiety/depression, and stroke. The selection process for the final analytical sample is presented in Figure 1.

### Dietary assessment

Dietary intake was assessed in the cohort 4 times up to age 53, and details about the dietary measures are available online [21]. At age 4, a single 24-h recall was completed by participants’ mothers or caretakers. The recall was completed during a home visit by an interviewer who asked, “What did this child have for each meal yesterday?” with prompted responses for breakfast, dinner, tea or high tea, and “last thing at night.” At ages 36 and 43, during interviews with research nurses, participants were asked to report all foods and drinks consumed in the past 2 d (i.e., 48-h recalls). They were then instructed to complete a diet diary for the subsequent 5 d [22]. At age 53, participants again completed 5-d food diaries but did not complete dietary recalls. For the diaries, participants were given detailed instructions to record all food and drinks, including alcohol, consumed throughout each of the 5 d. To further aid participants, a list of common foods and drinks was provided with descriptions, preparations, and amounts. At ages 43 and 53, participants were additionally given pictures to help indicate portion sizes.

The MRC used inhouse software, Diet In Nutrients Out (DINO), to code the dietary intakes, calculate consumption within food groups (e.g., total fruit, eggs), and derive nutrient data from relevant British food composition tables [23]. To facilitate our assessment of dietary patterns, we manually linked the cohort’s raw dietary data (excluding supplements) with foods from the 2015-2016 USDA Food Patterns Equivalents Database (FPED 1516) and Food Patterns Equivalents Ingredients Database (FPID 1516) using the closest matching item descriptions [24]. We calculated Pearson’s *r* correlations to examine agreement between NSHD and FPED components (i.e., food groups) based on our linkages (Supplemental Table 1).

From the linked NSHD and FPED dietary data, we calculated Healthy Eating Index (HEI)-2020 scores. Although this index was designed to assess dietary alignment with the Dietary Guidelines for Americans, 2020–2025 [25,26], it measures food groups thought to be culturally neutral and has been used to assess diet quality in other countries [27]. The HEI–2020 comprises 13 components, where 9 adequacy components (i.e., foods to prioritize) and 4 moderation components (i.e., foods to limit) are scored according to standards of higher or lower intake, respectively, per 1000 kcal of total energy in the diet [28]. The adequacy components include total vegetables, greens and beans, total fruit, whole fruits, whole grains, total dairy, total protein foods, seafood and plant proteins, and fatty acids ratio (MUFAs+PUFAs/SFAs). The moderation components are sodium, refined grains, saturated fats, and added sugars. Total HEI scores range from 0 to 100, with higher values indicating closer alignment with the dietary guidelines. To calculate HEI scores, we used the NIH National Cancer Institute’s (NCI) method for “describing dietary intake” [29]. Briefly, for multiple days of data reported in a single data collection period (e.g., 5-d food diaries), we summed the food patterns equivalents and nutrient values across all days per person. Being mindful of differences in nutrient profiles between foods in the United Kingdom and United States, we used nutrient data from NSHD (i.e., energy, sodium, fatty acids, and added sugars) and only the food group equivalent data from FPED and FPID (e.g., cup and ounce equivalents for fruit, dairy, nuts, etc.). In some data collection years, NSHD data showed added sugars equal to 0 g for foods categorized as pure sugars (e.g., honey, syrups, table sugar), so we corrected added sugars using NSHD data from previous years, or, if missing, we substituted FPED data. We, then, applied the HEI–2020 scoring algorithm to create mean component and total scores for each data collection year.

Three Plant-Based Diet Indexes (PDIs) were used to measure intake of plant-based foods: 1 for overall intake (PDI) and 1 each for intake of plant-based foods considered healthful (hPDI) or unhealthful (uPDI), depending on their associations with health outcomes like type 2 diabetes [30], coronary artery disease [31], and obesity [32]. For each index, plant- and animal-based components were either positively scored or reverse-scored per pre-established scoring algorithms [30,31]. Table 1 presents these algorithms and the 18 components comprising 7 healthy plant food groups (whole grains, fruits, vegetables, nuts, legumes, vegetables oils, and tea & coffee), 5 less healthy plant food groups (fruit juices, refined grains, potatoes, sugar-sweetened beverages, and sweets & desserts), and 6 animal food groups (animal fat, dairy, egg, fish or seafood, meat, and miscellaneous animal-based foods). Each component is given a score of 1–5 based on quintiles of intake, and total scores range from 18 to 90 for each index. In the overall PDI, all plant food groups are scored positively, and animal food groups are reverse-scored. For example, participants with the highest intakes of fish (quintile 5) are given a score of 1, and vice versa. For the hPDI, heathy plant food groups are scored positively, and all other food groups are reverse-scored. In the uPDI, less heathy plant food groups are scored positively, and all other food groups are reverse-scored. For all 3 indexes, higher total scores indicate lower intake of animal-based foods.

**TABLE 1:** Scoring algorithms for 18 plant- and animal-based dietary components in the 3 Plant-Based Diet Indexes

INDEX	POSITIVELY SCORED COMPONENTS	REVERSE-SCORED COMPONENTS	DESCRIPTION OF HIGH SCORES
	1–5 POINTS EACH	1–5 POINTS EACH	18–90 POINTS POSSIBLE
PDI	Healthy plant food groups: whole grains, fruits, vegetables, nuts, legumes, vegetable oils, and tea & coffee Less healthy plant food groups: fruit juices, refined grains, potatoes, sugar-sweetened beverages, and sweets & desserts	Animal food groups: animal fat, dairy, egg, fish or seafood, meat, and miscellaneous animal-based foods	High intake of healthy and less healthy plant food groups; low intake of animal food groups
hPDI	Healthy plant food groups: whole grains, fruits, vegetables, nuts, legumes, vegetable oils, and tea & coffee	Less healthy plant food groups: fruit juices, refined grains, potatoes, sugar-sweetened beverages, and sweets & desserts Animal food groups: animal fat, dairy, egg, fish or seafood, meat, and miscellaneous animal-based foods	High intake of healthy plant food groups; low intake of less healthy plant food groups and animal food groups
uPDI	Less healthy plant food groups: fruit juices, refined grains, potatoes, sugar-sweetened beverages, and sweets & desserts	Healthy plant food groups: whole grains, fruits, vegetables, nuts, legumes, vegetable oils, and tea & coffee Animal food groups: animal fat, dairy, egg, fish or seafood, meat, and miscellaneous animal-based foods	High intake of less healthy plant food groups; low intake of healthy plant food groups and animal food groups

hPDI, Healthful Plant-based Diet Index; PDI, Plant-based Diet Index; uPDI, Unhealthful Plant-based Diet Index.

In this study, dietary intakes for 15 of the PDI components were directly available or could easily be derived from NSHD raw dietary data. For the other 3 components (vegetable oil, animal fat, animal-based dairy), we relied on data from FPED or a mix of FPED and NSHD data using our previous database linkages. We estimated participants’ total vegetable oil intake using FPED “oils.” To estimate total animal fat intake, we subtracted NSHD “plant-based fats” quantities from FPED “solid fats.” To estimate animal-based dairy intake, we used FPED “total dairy” (which includes fortified soy beverages) minus NSHD “plant-based dairy” quantities. To create scores for the PDIs, we again utilized NCI’s method for “describing dietary intake” [29]. For multiple days of data reported in a single data collection period (e.g., 5-d food diaries), we summed intakes for the 18 components across all days per person. Histograms showed skewed distributions for several components, with some, like nuts and legumes, showing nearly 0 g of intake for many participants. To account for this, we adjusted intakes per 1000 kcal of total energy prior to determining quintiles of intake and applying scoring algorithms. Scores for the PDIs are typically based on absolute intakes, with total energy adjusted separately in statistical models [30], so we also created scores based on absolute intakes for use in sensitivity analyses as described further in the Supplemental Methods.

### Cognitive reserve

As explained above, premorbid cognitive abilities based on acquired knowledge and experiences, like reading and writing (crystallized abilities), are an indication of cognitive reserve [12]. Onset of Alzheimer’s dementia symptoms typically occurs after age 65 [33], so tests of reading and writing prior to symptom onset are used in clinical and research settings to indicate premorbid cognitive ability [12,13]. The NART is commonly used for this purpose, and at age 53, members of the 1946 British Birth Cohort were administered the NART by a research nurse during an in-person interview [34]. NART scores at this age are a suitable proxy for cognitive reserve because crystallized abilities typically peak in midlife and are relatively resilient to early changes [12], as evidenced by the relative stability of NART scores across participants aged 20–70 y [13]. The NART tests word pronunciation ability for 50 irregular words that are difficult to pronounce correctly based on spelling alone [35]. To generate NART scores, mispronounced words were tallied, and a count of correctly pronounced words was derived to create total scores ranging from 0 to 50, with higher scores indicating greater cognitive reserve.

### Covariates

In this ongoing cohort, participants contributed data 21 times from birth to age 53 [15]. Covariates of interest in the present study were grouped into sociodemographic factors, lifestyle factors, involvement in leisure activities, health outcomes, and childhood global cognitive ability. Sociodemographic factors were sex and region reported at birth, household social class (derived from father’s occupation) at participant age 11 (or if missing, age 15, then 4), highest level of education attempted or attained by age 43, and highest social class attained (derived from head of household’s occupation) by age 53. Lifestyle factors were cigarette status at age 53, quintiles of cumulative mean alcohol intake (g) to age 53, and leisure time physical activity at age 53. Involvement in other leisure activities was divided into intellectual and social categories as follows. At age 53, participants were asked about involvement and frequency of involvement in 1) constructive activities (e.g., making things) and 2) musical, artistic, and creative activities. We combined these 2 questions to derive number of activity types (none, 1, or 2) reported at age 53. The only social activity measured at age 53 was seeing friends or relatives once a month or more. We dichotomized this to indicate those who reported seeing zero versus 1 or more friends or relatives monthly. Health outcomes included in analyses were BMI, calculated from height and weight measured by registered nurses at age 53 (or if missing, age 43), and 6 self-reported health conditions at age 53: diabetes diagnosis (type not specified), severe headaches or migraines in the last 10 y, heart attack, high blood pressure in the last 10 y, reported use of epilepsy medication, and stroke in the last 10 y. Many participants did not report on nervous/emotional trouble or anxiety/depression at age 53, so we created a binary indicator of whether these conditions were never versus ever reported from age 26 to 53. Childhood cognitive ability at age 8 was assessed with 4 different tests: picture intelligence, reading comprehension, word reading, and vocabulary. Detailed descriptions of these measures were previously published [34]. Within the analytical sample, we created standardized scores for each measure of cognitive ability using a modified method that is robust to outlier influences (e.g., modified *z*-scores) [36]. We then summed the standardized scores to create a total score indicating global cognitive ability at age 8. For participants missing scores (*n* = 186; 7.4% of sample), we imputed the sample mean.

### Statistical analyses

Baseline comparisons between included and excluded participants were conducted with Pearson’s chi-square tests (χ^2^; for categorical variables) and independent means *t*-tests with unequal variances assumed (for continuous variables). Results with *P* < 0.05 were deemed statistically significant. Effect sizes were calculated using Cramér's V (categorical variables), where results were interpreted as weak (V > 0.05–0.1), moderate (V > 0.1–0.15), strong (V > 0.15–0.25), and very strong (V > 0.25), and using Cohen’s *d* for 2 independent samples (*d*_*s*_; continuous variables), where results were interpreted as small (*d*_*s*_ = |0.2|), medium (*d*_*s*_ = |0.5|), and large (*d*_*s*_ = |0.8|) according to common interpretations [37,38].

To determine typical dietary patterns over time, we calculated cumulative mean scores for the HEI and PDIs for participants with dietary data from ≥2 data collection periods. Dietary data were analyzed regardless of the number of dietary intake days reported in a given data collection period. Notably, except for *n* = 11 at age 43 and *n* = 3 at age 53, all participants in the analytical sample reported ≥2 d of dietary data at each data collection period (other than at age 4 when only 1 24-h recall was collected). For each dietary index, we divided participants into quintiles based on their cumulative mean scores. We then created radar plots depicting dietary intake for components of the HEI and PDIs across quintiles for these indexes. As above, we also conducted chi-square tests and calculated Cramér's V to compare categorical characteristics of the analytical sample across HEI-2020 quintile groups. For continuous measures, we compared HEI-2020 quintile groups using one-way analysis of variance (ANOVA) tests, where resulting *R*^2^ values (equivalent to η^2^) indicated effect sizes that were small (*R*^2^ = 0.01), medium (*R*^2^ = 0.06), and large (*R*^2^ = 0.14) based on conventions [38].

To examine associations between cognitive reserve and different dietary patterns, we first conducted unadjusted linear regression using dietary index quintiles as the model’s primary predictor (Q1 as the reference level) and continuous NART scores as the primary outcome (Model 1). Next, we conducted a series of multiple linear regression models, each with 1 additional covariate group as follows: Model 2 added sociodemographic factors, Model 3 added lifestyle factors, Model 4 added leisure activities, Model 5 added health outcomes, and Model 6 added childhood cognitive ability. Due to potential contributions of early-life diet to early-life cognition, overadjusting could not be completely ruled out in Model 6, but Pearson’s *r* correlations between age 4 diet and age 8 cognitive ability confirmed weak associations (*r* range = −0.03 for uPDI to 0.11 for HEI). Therefore, we selected Model 5 as our primary model when presenting results. We created forest plots to visually present the adjusted mean differences and 95% confidence intervals (CI) from Model 5 for each dietary index, along with adjusted means and SE for NART scores when covariates were set to reference levels (categorical) or means (continuous). Additional details are provided in the Supplemental Methods.

Following the multiple linear regression models, we conducted Wald *F* tests to determine which covariate groups contributed significantly (*P* < 0.05) to the total variance explained (*R*^2^) when predicting NART scores. Here, sociodemographic factors were divided into childhood and adulthood measures to uniquely identify these life stage contributions to cognitive reserve. We then determined proportion of *R*^2^ contributed by each covariate group by calculating decomposition of *R*^2^ using the Owen value [39]. The proportion of variability in NART explained by covariate groups was calculated as total *R*^2^ multiplied by each group’s proportion of *R*^2^ explained.

The 2015 and 2020 HEI scoring algorithms are identical, so HEI-2020 component and total scores were created using SAS software (version 9.4, SAS Institute, Inc.) and the HEI-2015 Scoring Macro (version 1.0, 06/25/2017) provided by NIH’s National Cancer Institute [40]. All other data management and analyses were performed using Stata software (version 18.0 for Windows, StataCorp LLC).

## Results

### Participant characteristics

Out of the 5362 original members of the 1946 British Birth Cohort, 2687 were eligible for this study, 173 were excluded due to missing covariate data, and the 2514 remaining participants (48.21% male) were included in analyses. Comparisons between all included and excluded participants revealed significant differences in every early-life characteristic examined except age 4 hPDI, and uPDI scores (Supplemental Tables 2 and 3). Effect sizes suggested weak (V ≤ 0.8) or small effects (*d*_*s*_ ≤ 0.25) for all childhood measures but a moderate effect for highest education level attempted or achieved to age 26 (V = 0.14). A higher percentage of those included were at O level or above (e.g., good knowledge and understanding of a subject), whereas a higher percentage of those excluded were at the sub-GCE/sub-Burnham C level or below (e.g., basic knowledge and skills) [41]. This difference is partly explained by less available education data after age 15 for those excluded.

Characteristics for the overall analytical sample and across HEI–2020 quintiles are presented in Table 2. Those with the highest HEI–2020 scores were more likely to be female, be born in southeast England, be from intermediate or professional class households, and have higher general cognitive functionality at age 8. They were more likely to have attempted or attained A level or higher education by age 43 and be from households with intermediate or professional social class by age 53. At 53, they were also more likely to have been a “never smoker,” consumed >0–2 units/d of alcohol, been most active, and engaged in more intellectual activities (musical/artistic/creative and constructive). Their cumulative mean energy (kcal/d) and alcohol intake (g/d) were lower than all other quintiles, and they had the lowest average BMIs and highest average NART scores. Finally, they were less likely to have high blood pressure between ages 43 and 53, but were more likely to have ever reported nervous/emotional trouble or anxiety/depression between ages 26 and 53.

**TABLE 2:** Characteristics of participants from the 1946 British Birth Cohort included in the full analytical sample with comparisons across Healthy Eating Index-2020 quintiles^1^

Characteristics	Full sample (*n* = 2514)	Healthy Eating Index (HEI)-2020 quintiles						
		Q1 (*n* = 503)	Q2 (*n* = 503)	Q3 (*n* = 503)	Q4 (*n* = 503)	Q5 (*n* = 502)		
	HEI range: 26.40–72.82	HEI range: 26.40–43.33	HEI range: 43.34–47.60	HEI range: 47.61–51.52	HEI range: 51.53–56.25	HEI range: 56.25–72.82		
Categorical characteristics^2^							χ^*2*^	V

Sex at birth							147.36^3^	0.24
Male	1212 (48.21)	312 (62.03)	298 (59.24)	254 (50.50)	198 (39.36)	150 (29.88)		
Female	1302 (51.79)	191 (37.97)	205 (40.76)	249 (49.50)	305 (60.64)	352 (70.12)		
Region of birth							60.87^4^	0.08
Wales	137 (5.45)	36 (7.16)	27 (5.37)	32 (6.36)	21 (4.17)	21 (4.18)		
Northern	197 (7.84)	52 (10.34)	46 (9.15)	34 (6.76)	37 (7.36)	28 (5.58)		
Yorkshire and Humberside	217 (8.63)	44 (8.75)	45 (8.95)	51 (10.14)	45 (8.95)	32 (6.37)		
Northwest England	259 (10.30)	48 (9.54)	54 (10.74)	57 (11.33)	58 (11.53)	42 (8.37)		
East Midlands	183 (7.28)	32 (6.36)	42 (8.35)	30 (5.96)	36 (7.16)	43 (8.57)		
West Midlands	203 (8.07)	43 (8.55)	35 (6.96)	44 (8.75)	36 (7.16)	45 (8.96)		
East Anglia	89 (3.54)	20 (3.98)	15 (2.98)	12 (2.39)	18 (3.58)	24 (4.78)		
South East England	799 (31.78)	127 (25.25)	154 (30.62)	156 (31.01)	175 (34.79)	187 (37.25)		
South West England	158 (6.28)	26 (5.17)	30 (5.96)	34 (6.76)	35 (6.96)	33 (6.57)		
Scotland	272 (10.82)	75 (14.91)	55 (10.93)	53 (10.54)	42 (8.35)	47 (9.36)		
Household social class at 11 y							173.55^3^	0.13
Professional etc.	172 (6.84)	8 (1.59)	33 (6.56)	32 (6.36)	40 (7.95)	59 (11.75)		
Intermediate	522 (20.76)	72 (14.31)	76 (15.11)	120 (23.86)	107 (21.27)	147 (29.28)		
Skilled (nonmanual)	418 (16.63)	53 (10.54)	79 (15.71)	89 (17.69)	102 (20.28)	95 (18.92)		
Skilled (manual)	797 (31.70)	197 (39.17)	182 (36.18)	158 (31.41)	154 (30.62)	106 (21.12)		
Partly skilled	457 (18.18)	116 (23.06)	105 (20.87)	82 (16.3)	74 (14.71)	80 (15.94)		
Unskilled	148 (5.89)	57 (11.33)	28 (5.57)	22 (4.37)	26 (5.17)	15 (2.99)		
Highest education level attempted or achieved by 43 y							228.88^3^	0.15
No qualifications or none attempted	610 (24.26)	199 (39.56)	150 (29.82)	105 (20.87)	88 (17.5)	68 (13.55)		
Vocational only	210 (8.35)	66 (13.12)	43 (8.55)	42 (8.35)	35 (6.96)	24 (4.78)		
Sub-GCE or sub-Burnham C	103 (4.1)	24 (4.77)	23 (4.57)	26 (5.17)	14 (2.78)	16 (3.19)		
O level or equivalent	510 (20.29)	79 (15.71)	110 (21.87)	100 (19.88)	116 (23.06)	105 (20.92)		
A level or equivalent	455 (18.1)	76 (15.11)	93 (18.49)	89 (17.69)	87 (17.3)	110 (21.91)		
Burnham A2	220 (8.75)	28 (5.57)	34 (6.76)	49 (9.74)	53 (10.54)	56 (11.16)		
First degree or graduate equivalent	356 (14.16)	30 (5.96)	45 (8.95)	77 (15.31)	96 (19.09)	108 (21.51)		
Higher degree, masters	21 (0.84)	≤ 10	≤ 10	≤ 10	≤ 10	≤ 10		
Higher degree, doctorate	29 (1.15)	≤ 10	≤ 10	≤ 10	≤ 10	≤ 10		
Highest social class (head of household) by 53 y							241.65^3^	0.16
Professional etc.	532 (21.16)	46 (9.15)	82 (16.30)	113 (22.47)	137 (27.24)	154 (30.68)		
Intermediate	1154 (45.9)	182 (36.18)	224 (44.53)	245 (48.71)	250 (49.70)	253 (50.40)		
Skilled (nonmanual)	274 (10.90)	67 (13.32)	64 (12.72)	52 (10.34)	48 (9.54)	43 (8.57)		
Skilled (manual)	504 (20.05)	191 (37.97)	121 (24.06)	82 (16.3)	61 (12.13)	49 (9.76)		
Partly skilled	44 (1.75)	13 (2.58)	11 (2.19)	11 (2.19)	≤ 10	≤ 10		
Unskilled	≤10	≤ 10	≤ 10	≤ 10	≤ 10	≤ 10		
Armed forces	≤10	≤ 10	≤ 10	≤ 10	≤ 10	≤ 10		
Cigarette status at 53 y							177.24^3^	
Current smoker	555 (22.08)	200 (39.76)	132 (26.24)	102 (20.28)	73 (14.51)	48 (9.56)		
Ex-smoker	892 (35.48)	169 (33.60)	176 (34.99)	177 (35.19)	187 (37.18)	183 (36.45)		
Never smoked	1067 (42.44)	134 (26.64)	195 (38.77)	224 (44.53)	243 (48.31)	271 (53.98)		
Alcohol (mean units/d) at 53 y							70.54^3^	
0	361 (23.01)	90 (39.65)	46 (17.49)	72 (21.88)	84 (23.40)	69 (17.65)		
>0–2	655 (41.75)	71 (31.28)	104 (39.54)	135 (41.03)	154 (42.90)	191 (48.85)		
>2–4	311 (19.82)	33 (14.54)	53 (20.15)	61 (18.54)	81 (22.56)	83 (21.23)		
>4	242 (15.42)	33 (14.54)	60 (22.81)	61 (18.54)	40 (11.14)	48 (12.28)		
Physical activity status at 53 y							209.87^3^	
Inactive	1203 (47.85)	327 (65.01)	281 (55.86)	229 (45.53)	215 (42.74)	151 (30.08)		
Less active	455 (18.10)	73 (14.51)	83 (16.50)	100 (19.88)	103 (20.48)	96 (19.12)		
Most active	856 (34.05)	103 (20.48)	139 (27.63)	174 (34.59)	185 (36.78)	255 (50.80)		
Leisure activities at 53 y								
Intellectual: musical/artistic/creative or constructive							90.78^3^	0.13
None	961 (38.23)	221 (43.94)	231 (45.92)	190 (37.77)	185 (36.78)	134 (26.69)		
One activity type	811 (32.26)	181 (35.98)	155 (30.82)	171 (34.00)	154 (30.62)	150 (29.88)		
Both activity types	742 (29.51)	101 (20.08)	117 (23.26)	142 (28.23)	164 (32.60)	218 (43.43)		
Social: Friends/relatives seen at least once a month							9.58^5^	0.06
None	89 (3.54)	12 (2.39)	28 (5.57)	20 (3.98)	15 (2.98)	14 (2.79)		
1 or more	2425 (96.46)	491 (97.61)	475 (94.43)	483 (96.02)	488 (97.02)	488 (97.21)		
Health status^6^ self-reported at 53 y								
Diabetes diagnosis	61 (2.43)	14 (2.78)	13 (2.58)	12 (2.39)	11 (2.19)	11 (2.19)	0.57	0.02
Severe headaches or migraines in last 10 y	568 (22.59)	110 (21.87)	103 (20.48)	107 (21.27)	120 (23.86)	128 (25.50)	4.82	0.04
Heart attack	39 (1.55)	≤ 10	12 (2.39)	≤ 10	≤ 10	≤ 10	4.01	0.04
High blood pressure in last 10 y	482 (19.17)	112 (22.27)	109 (21.67)	99 (19.68)	77 (15.31)	85 (16.93)	11.69^5^	0.07
Epilepsy medication use	29 (1.15)	≤ 10	≤ 10	≤ 10	≤ 10	≤ 10	0.49	0.01
Nervous/emotional trouble or anxiety/depression, 26–53 y	1022 (40.65)	168 (33.40)	169 (33.60)	200 (39.76)	222 (44.14)	263 (52.39)	52.70^3^	0.14
Stroke in last 10 y	20 (0.80)	≤ 10	≤ 10	≤ 10	≤ 10	≤ 10	1.52	0.02

Continuous characteristics							*F*	

Energy (kcal), cumulative mean by 53 y								
Mean (SD)	1845.34 (403.71)	1942.35 (452.74)	1904.52 (404.62)	1858.29 (387.26)	1810.27 (371.07)	1710.97 (356.94)	25.93^3^	
Median (IQR)	1811.87 (546.87)	1926.93 (655.05)	1883.16 (531.55)	1833.97 (558.03)	1783.79 (484.15)	1671.79 (459.94)		
Alcohol intake (g/d), cumulative mean by 53 y								
Mean (SD)	220.47 (371.52)	263.69 (437.52)	297.39 (450.58)	239.35 (403.33)	160.31 (249.99)	141.43 (231.11)	16.81^3^	
Median (IQR)	83.29 (241.54)	86.95 (374.03)	122.47 (346.86)	86.32 (262.94)	81.93 (179.11)	70.45 (133.66)		
BMI (kg/m^2^) at 53 y (if missing, 43 y)								
Mean (SD)	27.37 (4.81)	28.11 (4.76)	27.46 (5.02)	27.26 (4.66)	27.13 (4.78)	26.91 (4.73)	4.54^4^	
Median (IQR)	26.60 (5.69)	27.68 (5.65)	26.58 (5.72)	26.60 (5.89)	26.21 (5.61)	25.90 (5.53)		
Global cognitive ability (robust *z*-scores) at 8 y^7^								
Mean (SD)	−0.14 (3.10)	−1.29 (3.03)	−0.88 (2.85)	0.15 (3.02)	0.37 (2.95)	0.94 (3.09)	47.5^3^	
Median (IQR)	−0.14 (4.17)	−1.15 (3.91)	−0.56 (3.76)	−0.04 (4.20)	0.11 (3.94)	0.90 (3.46)		
NART scores (0–50 possible) at 53 y								
Mean (SD)	34.34 (9.48)	29.80 (9.66)	32.63 (9.10)	34.89 (9.33)	36.46 (8.70)	37.94 (8.33)	63.49^3^	
Median (IQR)	36 (14)	31 (14)	34 (13)	36 (13)	38 (12)	40 (10)		

A level, advanced level; GCE, General Certificate of Education; NART, National Adult Reading Test; O level, ordinary level.

1 Comparisons were made using Pearson’s chi-square tests (χ^2^) with Cramér's V effect sizes for categorical characteristics, and one-way analysis of variance (ANOVA) *F* tests for continuous characteristics.

2 *n* (%); To protect the identity of cohort members in this ongoing study, data were suppressed in cells where *n* ≤10.

3 *P* < 0.001.

4 *P* < 0.01.

5 *P* < 0.05.

6 For health status categories, comparisons were for those who reported having or not having the outcome.

7 If missing, sample mean was imputed. The number of imputations did not differ between quintile groups (χ^2^ = 4.55, *P* = 0.34, V = 0.04).

Cumulative mean scores for all dietary indexes were calculated from dietary data collected at ages 4, 36, 43, and 53. The number of participants in the analytical sample reporting data at each age (i.e., data collection period) and the mean number of days reported are presented in Supplemental Table 4. Nearly all participants contributed data at ages 4 (97.7%) and 43 (96.3%), whereas 49.3% contributed data at all 4 time points (31.8% contributed 3 times; 18.9% contributed 2 times; data not shown). Unadjusted median HEI-2020 scores increased in stepwise fashion over time, whereas scores for all 3 plant-based indexes either increased (PDI) or decreased (hPDI and uPDI) from age 4 to 36, then plateaued (see Supplemental Figures 1–4). Unadjusted averages for dietary index cumulative mean scores for the analytical sample overall and by quintiles are presented in Table 3. HEI–2020 scores ranged from 26.4 to 72.8 with an overall mean (SD) of 49.9 (7.54). For the Plant-Based Diet Indexes, overall mean (SD) scores suggested higher intakes of less healthy plant foods in the sample (uPDI = 56.3 ± 4.6, range: 40.3–70.0) compared with overall and healthy plant-based foods (PDI = 52.0 ± 4.4, range: 37.0–66.3; hPDI = 54.0 ± 3.8, range: 40.0–67.3). Radar plots of component scores (unadjusted medians) for the HEI–2020 and PDIs are presented in Supplemental Figures 5–8.

**TABLE 3:** Unadjusted averages for dietary index cumulative mean scores from the full analytical sample and by dietary index quintiles

Index	Full sample	Q1	Q2	Q3	Q4	Q5
HEI-2020 (0–100 possible)						
Sample size, *n*	2514	503	503	503	503	502
Mean (SD)	49.86 (7.54)	39.61 (3.01)	45.55 (1.28)	49.57 (1.10)	53.81 (1.33)	60.76 (3.58)
Median (IQR)	49.58 (10.48)	40.29 (3.86)	45.54 (2.26)	49.58 (1.82)	53.81 (2.25)	59.88 (4.87)
PDI (18–90 possible)						
Sample size, *n*	2514	535	506	480	544	449
Mean (SD)	52.01 (4.44)	45.82 (2.25)	49.96 (0.72)	52.25 (0.61)	54.69 (0.84)	58.35 (1.83)
Median (IQR)	52.25 (6.00)	46.33 (3.17)	50 (1.17)	52.25 (1.00)	54.67 (1.50)	58 (2.50)
hPDI (18–90 possible)						
Sample size, *n*	2514	561	515	445	502	491
Mean (SD)	53.95 (3.84)	48.88 (1.79)	52.17 (0.56)	54.00 (0.48)	55.98 (0.65)	59.47 (1.89)
Median (IQR)	53.75 (5.00)	49.25 (2.50)	52.25 (1.00)	54 (0.67)	56 (1.00)	59 (2.50)
uPDI (18–90 possible)						
Sample size, *n*	2514	514	518	510	475	497
Mean (SD)	56.30 (4.58)	49.91 (2.22)	54.01 (0.78)	56.43 (0.68)	58.82 (0.74)	62.74 (2.07)
Median (IQR)	56.25 (6.08)	50.5 (2.92)	54 (1.33)	56.5 (1.00)	58.75 (1.25)	62.25 (2.75)

HEI, Healthy Eating Index; hPDI, Healthful Plant-based Diet Index; PDI, Plant-based Diet Index; Q, quintile; uPDI, Unhealthful Plant-based Diet Index.

### Dietary pattern indexes and cognitive reserve

**FIGURE 2:**
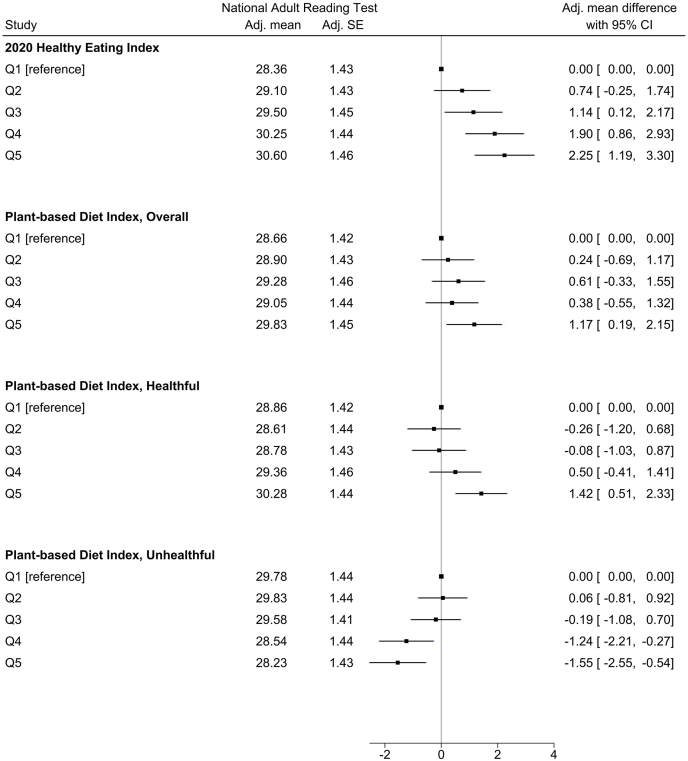
Forest plot of NART scores by dietary index quintiles. Effect sizes presented are from adjusted multiple linear regression models (Model 5) with robust SEs; Covariates included sex, region of birth, cigarette status, alcohol intake, exercise status, intellectual activities, social activities, BMI, diabetes, headaches/migraines, heart attack, high blood pressure, epilepsy medication, nervous/emotional trouble or anxiety/depression, and stroke. Adjusted means and SE represent NART scores when covariates are set to reference levels (categorical) and means (continuous). NART, National Adult Reading Test.

Extreme quintiles of cumulative mean dietary index scores showed significant associations with NART scores in adjusted multiple linear regression models, and results are presented in Table 4. Overall, Models 1 through 6 indicated a potential dose-response relationship between NART scores and scores on the HEI–2020, hPDI, and uPDI. Mean NART scores generally increased in stepwise fashion across quintiles for HEI and hPDI and decreased in stepwise fashion across quintiles for uPDI. For the overall PDI, NART scores also tended to increase across quintiles, but no clear dose-response pattern was identified. These trends are depicted in Figure 2, which presents results from Model 5, the main model. Although adjusting for sociodemographic factors, lifestyle factors, leisure activities, and health outcomes, HEI–2020 Quintiles 3 through 5 were associated with significantly higher NART scores compared with quintile 1 [adjusted mean difference (95% CI) for Q3: 1.14 (0.12, 2.17); Q4: 1.90 (0.86, 2.93); and Q5: 2.25 (1.19, 3.30)]. For the PDI and hPDI, only the highest intakes of plant-based foods were associated with significantly higher NART scores compared with quintile 1 [PDI Q5: 1.17 (0.19, 2.15); hPDI Q5: 1.42 (0.51, 2.33)]. Conversely, uPDI Quintiles 4 and 5, with the highest intakes of less healthy plant-based foods, were associated with significantly lower NART scores compared with quintile 1 [Q4: −1.24 (−2.21, −0.27); Q5: −1.55 (−2.55, −0.54)]. For each dietary index, β-coefficients and 95% CI for all Model 5 covariates are presented in Supplemental Table 5. Findings from sensitivity analyses using plant-based diet index scores based on absolute intakes, with models separately adjusted for total energy (kcal), showed the same trends as the original models (Supplemental Methods and Supplemental Table 6).

**TABLE 4:** Results from linear regression models^1^ for NART scores across different dietary index quintiles with covariate groups^2^ added to each model (*n* = 2514)

Index	Model 1 Unadjusted	Model 2 1 + Sociodem.	Model 3 2 + Lifestyle	Model 4 3 + Leisure activities	Model 5 4 + Health status	Model 6 5 + Childhood cog.
	*β* (95% CI)	*β* (95% CI)	*β* (95% CI)	*β* (95% CI)	*β* (95% CI)	*β* (95% CI)
HEI-2020, *R*^2^	**0.0919**	**0.3855**	**0.3956**	**0.4003**	**0.4038**	**0.5188**
Q2	2.83 (1.67, 3.99)	0.84 (−0.61, 1.84)	0.68 (−0.32, 1.67)	0.72 (−0.28, 1.71)	0.74 (−0.25, 1.74)	0.94 (0.05, 1.84)
Q3	5.09 (3.92, 6.27)	1.46 (0.43, 2.49)	1.15 (0.12, 2.18)	1.13 (0.11, 2.15)	1.14 (0.12, 2.17)	0.70 (−0.21, 1.62)
Q4	6.66 (5.52, 7.79)	2.29 (1.26, 3.32)	1.91 (0.86, 2.96)	1.91 (0.87, 2.95)	1.90 (0.86, 2.93)	1.48 (0.57, 2.40)
Q5	8.14 (7.02, 9.25)	2.83 (1.79, 3.86)	2.32 (1.27, 3.38)	2.23 (1.18, 3.28)	2.25 (1.19, 3.30)	1.53 (0.59, 2.46)
PDI, *R*^2^	**0.0146**	**0.3789**	**0.3917**	**0.3966**	**0.4001**	**0.5173**
Q2	0.64 (−0.52, 1.80)	0.37 (−0.56, 1.30)	0.25 (−0.68, 1.18)	0.25 (−0.68, 1.18)	0.24 (−0.69, 1.17)	0.37 (−0.47, 1.20)
Q3	1.92 (0.75, 3.09)	0.82 (−0.10, 1.75)	0.69 (−0.25, 1.63)	0.66 (−0.28, 1.59)	0.61 (−0.33, 1.55)	0.78 (−0.05, 1.61)
Q4	1.93 (0.77, 3.08)	0.56 (−0.37, 1.48)	0.45 (−0.49, 1.38)	0.43 (−0.50, 1.36)	0.38 (−0.55, 1.32)	0.37 (−0.46, 1.20)
Q5	3.35 (2.19, 4.52)	1.45 (0.47, 2.43)	1.27 (0.28, 2.26)	1.19 (0.21, 2.17)	1.17 (0.19, 2.15)	0.99 (0.12, 1.85)
hPDI, *R*^2^	**0.0302**	**0.3819**	**0.3944**	**0.3988**	**0.4022**	**0.5183**
Q2	0.72 (−0.43, 1.87)	−0.16 (−1.11, 0.78)	−0.27 (−1.21, 0.68)	−0.30 (−1.24, 0.64)	−0.26 (−1.20, 0.68)	−0.25 (−1.09, 0.59)
Q3	1.53 (0.35, 2.71)	−0.09 (−1.06, 0.88)	−0.09 (−1.05, 0.88)	−0.06 (−1.01, 0.90)	−0.08 (−1.03, 0.87)	−0.22 (−1.08, 0.64)
Q4	2.94 (1.82, 4.05)	0.73 (−0.19, 1.65)	0.52 (−0.40, 1.44)	0.51 (−0.41, 1.42)	0.50 (−0.41, 1.41)	0.50 (−0.30, 1.30)
Q5	4.56 (3.47, 5.64)	1.73 (0.82, 2.65)	1.58 (0.66, 2.49)	1.43 (0.52, 2.34)	1.42 (0.51, 2.33)	0.98 (0.16, 1.79)
uPDI, *R*^2^	**0.0420**	**0.3821**	**0.3946**	**0.3991**	**0.4027**	**0.5197**
Q2	−0.98 (−2.05, 0.09)	−0.08 (−0.95, 0.79)	0.01 (−0.86, 0.87)	0.08 (−0.78, 0.94)	0.06 (−0.81, 0.92)	0.51 (−0.27, 1.29)
Q3	−2.10 (−3.17, −1.03)	−0.39 (−1.29, 0.51)	−0.30 (−1.19, 0.59)	−0.19 (−1.08, 0.70)	−0.19 (−1.08, 0.70)	0.38 (−0.43, 1.19)
Q4	−4.03 (−5.21, −2.85)	−1.45 (−2.42, −0.49)	−1.34 (−2.30, −0.38)	−1.22 (−2.19, −0.25)	−1.24 (−2.21, −0.27)	−0.81 (−1.69, 0.07)
Q5	−5.29 (−6.43, −4.15)	−1.90 (−2.90, −0.91)	−1.68 (−2.68, −0.69)	−1.52 (−2.52, −0.52)	−1.55 (−2.55, −0.54)	−0.95 (−1.85, −0.04)

HEI, Healthy Eating Index; hPDI, Healthful Plant-based Diet Index; NART, National Adult Reading Test; PDI, Plant-based Diet Index; Q, quintile; uPDI, Unhealthful Plant-based Diet Index.

1 Mean differences (beta coefficients), 95% CIs, and *R*^2^ results are presented from linear regression models with robust standard errors and quintile 1 as the reference group.

2 Covariates for each model are as follows. Model 1: Unadjusted (dependent variable = NART scores; independent variables = dietary index quintiles); Model 2: Model 1 plus all sociodemographic factors (Childhood: sex, region of birth, social class; Adulthood: highest education level, highest household social class); Model 3: Model 2 plus lifestyle factors (cigarette status, alcohol cumulative mean quintiles, exercise status); Model 4: Model 3 plus leisure activity factors (intellectual and social activities); Model 5: Model 4 plus health status measures (BMI, diabetes, headache/migraine, heart attack, high blood pressure, epilepsy medication use, nervous condition, stroke); Model 6: Model 5 plus childhood cognitive ability.

### Covariate group contributions to variance explained

**FIGURE 3:**
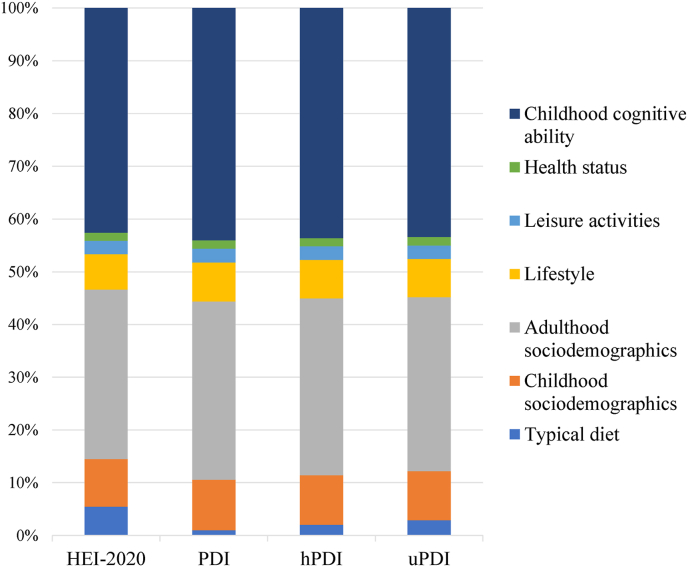
Percentage of the total variance explained by 7 covariate groups adjusted in multiple linear regression models. *R*^2^ = 0.52 for each model. HEI, Healthy Eating Index; hPDI, Healthful Plant-based Diet Index; PDI, Plant-based Diet Index; uPDI, Unhealthful Plant-based Diet Index.

**FIGURE 4:**
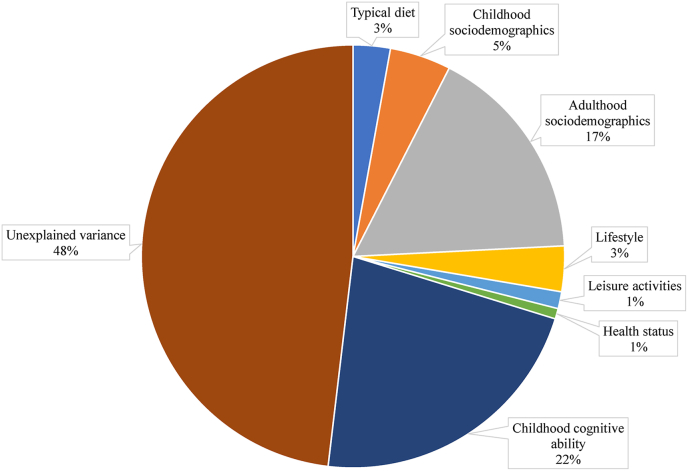
Percentage of the total variability in NART scores unexplained and explained by 7 covariate groups adjusted in multiple linear regression models, where “typical diet” was based on Healthy Eating Index-2020 quintiles. NART, National Adult Reading Test.

In the fully adjusted multiple linear regression models (Model 6), which adjusted for childhood cognitive ability, the covariates explained 52% of the variability in NART scores, as seen in Table 5. Figure 3 depicts the proportion of *R*^2^ contributed by each covariate group for each dietary index model, and Figure 4 depicts the proportion of total variability in NART scores explained by covariate groups or unexplained by the model when traditional diet was based on HEI–2020 quintiles. Results from Wald *F* tests showed that primary predictors, HEI, hPDI, uPDI, and all other covariate groups contributed significantly to these models (*P* < 0.05 for all groups). The one exception was PDI (*F*_4, 2463_ = 1.56, *P* = 0.18), which did not contribute significantly to variance explained. Among the dietary indexes, HEI–2020 scores contributed the most to total variance explained (5.47%), followed by uPDI (2.90%), hPDI (2.02%), and then PDI (0.99%). The percent contribution to total variability in NART followed the same trend: HEI (2.84%), uPDI (1.51%), hPDI (1.05%), and PDI (0.51%).

**TABLE 5:** Proportion of total variance explained (*R*^2^) in the model and of variation in NART scores explained by covariate groups^1^ in fully adjusted multiple linear regression models with different dietary indexes as primary predictors (*n* = 2514)

Covariate groups	Percent of total variance explained in models				Percent of total variation in NART explained			
	HEI-2020	PDI	hPDI	uPDI	HEI-2020	PDI	hPDI	uPDI
	*R*^2^ = 0.5188	*R*^2^ = 0.5173	*R*^2^ = 0.5183	*R*^2^ = 0.5197	51.88%	51.73%	51.83%	51.97%
Typical diet	5.47	0.99^2^	2.02	2.90	2.84	0.51^2^	1.05	1.51
Childhood sociodemographics	9.00	9.59	9.43	9.28	4.67	4.96	4.89	4.82
Adulthood sociodemographics	32.16	33.81	33.50	33.01	16.69	17.49	17.36	17.16
First degree or higher	9.83	10.28	10.17	10.10	5.10	5.32	5.27	5.25
Skilled (manual) social class	7.74	8.36	8.23	7.98	4.02	4.32	4.27	4.15
Lifestyle	6.72	7.36	7.34	7.29	3.48	3.81	3.81	3.79
Leisure activities	2.49	2.63	2.54	2.52	1.29	1.36	1.32	1.31
Health status	1.54	1.57	1.54	1.62	0.80	0.81	0.80	0.84
Childhood cognitive ability	42.61	44.04	43.63	43.38	22.11	22.78	22.61	22.54
Diet as a lifestyle factor								
Lifestyle + typical diet^3^	10.92	8.10	9.05	9.81	5.67	4.19	4.69	5.10

HEI, Healthy Eating Index; hPDI, Healthful Plant-based Diet Index; NART, National Adult Reading Test; PDI, Plant-based Diet Index; uPDI, Unhealthful Plant-based Diet Index.

1 Covariate groups and included covariates: Typical diet (dietary index quintiles); Childhood sociodemographic factors (sex, region of birth, childhood social class); Adulthood **S**ociodemographic factors (highest education level and highest household social class); Lifestyle factors (cigarette status, alcohol cumulative mean quintiles, exercise status); Leisure activities (intellectual and social activities); Health status (BMI, diabetes, headache/migraine, heart attack, high blood pressure, epilepsy medication use, nervous condition, stroke); Childhood cognitive ability (sum of 4 cognitive measures at age 8).

2 *P* > 0.05 from Wald *F* tests (*P* < 0.05 for all others).

3 Proportion of variance explained when typical diet is grouped with other lifestyle factors. Note: This causes all other covariate group values to change (data not shown).

The covariate with the single greatest explanatory power was childhood global cognitive ability at age 8, which accounted for >40% of *R*^2^ in the fully adjusted models. The next greatest contribution was by adulthood sociodemographic factors (highest education level attempted or attained and highest household social class) together accounting for >30% of *R*^2^, where the most explanatory power came from the “1^st^ degree or higher” education level (∼10% of *R*^2^) and “skilled (manual)” social class (∼8% of *R*^2^). The other covariate groups each contributed <10% to total variance explained (childhood sociodemographic factors, 9% to 9.6%; lifestyle, 6.7% to 7.4%; leisure activities, 2.5% to 2.6%; and health status, 1.5% to 1.6%). When typical dietary pattern was grouped with other lifestyle factors, the group contribution to *R*^2^ ranged from 8.1% (PDI model) to 10.9% (HEI model).

## Discussion

This study investigated the association between alignment with different dietary patterns and cognitive reserve, indicated by NART scores at age 53, in the 1946 British Birth Cohort. The findings supported our hypothesis that greater alignment with healthy dietary patterns (i.e., higher HEI, PDI, and hPDI scores) would be positively associated with cognitive reserve, whereas greater alignment with less healthy dietary patterns (i.e., higher uPDI scores) would be inversely associated with cognitive reserve. In particular, participants whose typical dietary patterns 1) were most consistent with guidance from the Dietary Guidelines for Americans (HEI Q5), 2) had the highest intakes of plant food groups overall (PDI Q5), and 3) had the highest intakes of healthy plant food groups in particular (hPDI Q5), all had significantly higher cognitive reserve at age 53 compared with participants with opposing dietary patterns (Q1 for each index). Participants with dietary patterns most aligned with an unhealthful plant-based dietary pattern (uPDI Q5) had lower cognitive reserve than those least aligned (uPDI Q1). This hypothesis was further supported in that dose-response relationships were identified for the HEI-2020, hPDI, and uPDI when adjusting for important covariate groups. Together, these findings support recommendations to follow a healthy dietary pattern that includes healthy plant-based food groups and limits less healthy plant-based food groups. The findings also suggest the importance of a healthy diet in childhood and throughout adulthood for maintaining cognitive health across the lifespan.

Results from this study also indicated that HEI–2020, hPDI, and uPDI dietary patterns each uniquely and significantly explained total variability in cognitive reserve in addition to what was explained by other major contributing factors like childhood cognitive ability, highest education level, and highest social class. However, overall PDI did not contribute significantly to total variance explained in our models, which is not completely surprising. This finding may indicate that associations between plant-based dietary patterns and cognitive reserve depend on the quality of foods selected (e.g., the healthy and less healthy plant-based food groups) rather than the mere quantity of plant foods in the diet. This is supported by guidance in the Dietary Guidelines for Americans to prioritize whole grains, whole fruits, and whole vegetables in the diet and to limit intakes of refined grains, sugar-sweetened juices, and vegetables in the form of salty snacks [26].

Our findings were similar to those of a cross-sectional analysis of the Cognitive Function and Ageing Study Wales (CFAS-Wales) cohort (*n* = 2315) which found positive and significant associations for healthy diet with cognitive reserve after controlling for age, sex, chronic conditions, cognitive and social activity, and lifestyle [42]. Compared with our findings, CFAS-Wales results indicated a stronger association between healthy diet and cognitive reserve (*β* = 0.21; 95% CI: 0.16, 0.25), and this association was virtually unchanged from their unadjusted to fully adjusted models. This difference may be explained by their study measures and analysis methods. A cognitive reserve score was calculated from 2 proxy measures, educational level and occupational complexity, as is often done [4]. Investigators also developed an inhouse score for healthy diet based on a single food frequency questionnaire, and diet was modeled as a continuous measure. Finally, the CFAS-Wales is a cohort aged ≥65 y, so early-life environment/circumstances (e.g., childhood social class in our study) and cognitive ability were not accounted for. Our findings revealed that childhood cognitive ability was the single greatest contributor to variance explained in cognitive reserve at age 53, and with each additional covariate group added to our unadjusted models, the magnitudes of association and variance explained by dietary patterns were both greatly reduced. This suggests that, although dietary patterns should be included among the list of factors contributing to cognitive reserve, diet’s unique explanatory power may be limited. However, when combined with other lifestyle factors, diet helped explain roughly 5% of total variability in cognitive reserve, which is similar to the explanatory power of achieving a first degree or higher in education.

Analyses of truly longitudinal cohorts incorporating the complexities of these factors across the life course are needed to clarify the potential role of diet in the development of crystallized abilities and levels of peak premorbid cognitive ability, which represents cognitive reserve. Studies that include measures of both diet and cognition from early to later life will need to examine the correlations between these measures and carefully plan statistical models to minimize or avoid overadjustment. Research has shown that early-life nutrition is essential for early brain development and that the effect of malnutrition on neurodevelopment in early-life can have a lifelong impact [43]. In the present study, dietary intake at age 4 showed a weak correlation with childhood cognitive ability at age 8, so we could not rule out potential contributions of early-life diet to childhood cognitive ability. because age 4 diet was included in the cumulative diet exposure, our fully adjusted model risked overadjustment. Therefore, we conservatively chose the model without childhood cognitive ability as the primary model. In other studies on cognitive reserve that lack early-life dietary data, adjusting for childhood cognitive ability may be advised because it is a major contributor to cognition in later life.

Despite finding support for our hypothesis, the different magnitudes of association for the HEI compared with the 3 plant-based indexes were somewhat surprising. These differences may be partly explained by the HEI’s positive scoring, in contrast with the PDIs’ negative scoring, of fish and other animal-based foods that may contribute to cognitive health. Fatty fish in particular is a major dietary source of essential ω-3 fatty acids, which help form brain cell membranes and enable proper neuronal functioning [9]. In a systematic review comparing Mediterranean (MedDi), Dietary Approaches to Stop Hypertension (DASH), and Mediterranean-DASH Intervention for Neurodegenerative Delay (MIND) Diets, fish consumption was associated cross-sectionally with a lower risk of dementia and better attention in some studies. However, the cross-sectional studies found no real effect on global cognition, measures of fluid intelligence (e.g., executive function, working memory, visual memory, episodic verbal memory), or mild cognitive impairment [44]. For diets that exclude fish and seafood, ω-3s are available from eggs [9] and numerous plant-based sources, including nuts, seeds, seed oils [45], oils from marine microalgae, cruciferous and leafy vegetables, beans, and whole grains [9]. Longitudinal studies in the systematic review showed mixed results for fish consumption and seed oils, but nut consumption in general was associated in some studies with better cognitive function and lower risk of cognitive impairment [44]. Although the impact of fish intake and ω-3s on cognitive reserve is unclear from these studies, the negative scoring of fish and seafood in the PDIs may overlook the positive contributions of these items to cognitive health, thus reducing their explanatory power in cognition-focused research.

The difference in magnitudes of association for the HEI and PDIs in our study could also be partly explained by the hPDI and uPDI scoring algorithms, which count all fruit juices and white potatoes, regardless of their form, among the less healthy food groups due to their positive associations with type 2 diabetes outcomes [30]. However, some forms of these food groups may be beneficial for cognitive outcomes. A systematic review found that consumption of 100% juice was associated with lower incidence of AD in longitudinal cohorts and verbal memory benefits for those with mild cognitive impairment in intervention studies [46]. Separately, a cross-sectional study reported consumption of potatoes ≤100–150 g/d showed a dose-response relationship with higher global cognition measured by the Mini-Mental State Examination [47]. Concerns about fruit juice tend to focus on the higher energy density, higher sugar content, and lower dietary fiber content per serving than whole fruits, but 100% fruit juices include no added sugar and contain naturally occurring flavonoids and vitamin C, a powerful antioxidant that can reduce inflammation [48,49]. Concerns about white potatoes focus on high starch content and certain preparations – primarily as savory snacks, in fried forms, and with additions like butter and salt [26], but potatoes are also good sources of dietary fiber, resistant starch, potassium, and vitamin C [50]. Due to these qualities, 100% fruit juices and potatoes can contribute to the total beneficial properties of plant-based food groups, which, as previously stated, help mitigate the oxidative stress, inflammation, and vascular risk factors associated with age-related cognitive impairment and disease [8]. In the 1946 British Birth Cohort, most fruit juice was consumed as pure fruit juices or smoothies, where the mean added sugar content across all reported fruit-based drinks was low (< 4 g), and 70% of reported potato-based foods were boiled, baked, mashed, or raw, whereas the other 30% were fried (e.g., chips and crisps). Lumping all juices and potatoes into the “less healthy” food groups may have weakened associations between the healthful and unhealthful plant-based dietary patterns and cognitive reserve. In accordance with the Dietary Guidelines for Americans, the HEI–2020 counts 100% fruit juices toward total fruits and potatoes toward starchy vegetables, which are both adequacy components [26,28]. The HEI’s ability to discern quality regardless of food source [51] may partly explain why the magnitude of association between HEI quintiles and cognitive reserve appeared much stronger than the hPDI and uPDI. Modified versions of these indexes should be assessed in relation to cognitive reserve in future studies.

Aside from these differences in categorizing foods, indexes used in this study can be further distinguished by their theoretical approaches. That is, the HEI is based on recommended thresholds of intake (per 1000 kcal) known to be beneficial for health [27,51], whereas the PDIs are based solely on relative intake ranks (quintiles) within the population of study, where intakes may not meet levels recommended for good health [30]. Our use of the energy-adjusted scoring method (rather than the more common absolute intake approach) enabled better comparisons between the plant-based indexes and the HEI. Yet in our models, the HEI–2020 scores were more strongly associated with cognitive reserve than the 3 PDIs, and the HEI also explained more variability in NART scores. These findings indicate that dietary intakes based on recommended thresholds, as in the Dietary Guidelines for Americans, may be better able to distinguish between low- and high-quality diets than simple measures of proportion of healthy or less healthy food groups, where a high score does not necessarily imply a healthful intake level.

### Strengths and limitations

The primary strength of this study came from the use of data collected over 5 decades in an ongoing birth cohort, which enabled the use of multiple repeated dietary measures and important factors relevant to cognitive reserve from different stages in life. Despite this, there are possible residual effects that may have affected cognitive reserve, so residual confounding cannot be ruled out. The primary limitation of our study was the use of a cross-sectional design. Longitudinal models are necessary to determine whether and how dietary patterns are associated with the development of cognitive reserve. However, our method of averaging across multiple years with dietary measures allowed us to investigate associations between cumulative diet and cognitive reserve, which is accumulated over decades and reaches a peak in mid- to upper midlife. Other strengths included the use of the NART, a reliable and stable measure of cognitive reserve, and the use of multiple dietary indexes to compare associations across different dietary patterns. The latter also introduced limitations, as the PDIs were originally developed with type 2 diabetes risk factors in mind, so hPDI and uPDI scoring algorithms did not reflect special considerations related to cognitive outcomes, as described above. Despite this, the generalizability of our findings was strengthened by our use of the NART and dietary indexes that have been applied in different populations, as well as a cohort designed to represent diverse regions across the United Kingdom and a range of socioeconomic backgrounds.

In conclusion, this study of the 1946 British Birth Cohort investigated associations between different dietary patterns and cognitive reserve. Findings suggest that healthy dietary patterns, especially diets aligned with the Dietary Guidelines for Americans and diets high in total and healthful plant-based food groups, were associated with higher cognitive reserve. Diets higher in less healthy plant-based food groups were associated with lower cognitive reserve. Healthy dietary patterns and those highest in healthy and less healthy plant-based food groups helped explain variability in cognitive reserve, suggesting that diet may be considered a significant contributing factor alongside well-known influences like childhood cognitive ability, education level, social class, and other lifestyle factors. Longitudinal analyses and examinations in other populations are needed to confirm these findings, and additional studies are needed to further understand the impact of dietary alignment with the PDI, hPDI, and uPDI on a variety of health outcomes.

## Author contributions

The authors’ responsibilities were as follows – KCC designed and conducted the research, analyzed the data, and wrote the article. MC, PFJ, and TMS advised KCC throughout the entire research process. KCC had primary responsibility for final content; and all authors: have read and approved the final manuscript.

## Data availability

Data described in the manuscript, code book, and analytic code will be made available to bona fide researchers upon request to the NSHD Data Sharing Committee via a standard application procedure. Further details can be found at https://nshd.mrc.ac.uk/data-sharing/.

## Funding

This study received no external funding. Tufts had no role in the study design; the collection, analysis, and interpretation of data; the writing of the manuscript; or the decision where to submit the article for publication.

## Conflict of interest

KCC was supported by a PhD stipend from the Gerald J. and Dorothy R. Friedman School of Nutrition Science and Policy at Tufts University. PFJ’s effort on this study was supported by the US Department of Agriculture Agricultural Research Service Cooperative Agreement #58-8050-9-004. The other authors have nothing to disclose.
