# Supplementary material for: Associations Between Healthy and Plant-Based Dietary Patterns and Cognitive Reserve: A Cross-Sectional Analysis of the 1946 British Birth Cohort
Source: Curr Dev Nutr. 2025 Nov 12;9(12):107599. doi: 10.1016/j.cdnut.2025.107599 (PMC12718185; doi:10.1016/j.cdnut.2025.107599)
Supplement: Multimedia component 1 [file mmc1.docx]

**Associations between healthy and plant-based dietary patterns and cognitive reserve: A cross-sectional analysis of the 1946 British Birth Cohort**

Authors: Kelly C. Cara, Tammy M. Scott, Paul F. Jacques, Mei Chung

**Supplemental Methods**

For each dietary index, the fully adjusted multiple linear models were checked for collinearity and violations of linearity, homoscedasticity, and normality assumptions. No collinearity was detected between the one continuous covariate (BMI) and NART scores based on a priori standards of variance inflation factors < 6. Visual assessment of component plus residual (CPR) plots indicated linearity was not violated. Homoscedasticity was checked with two-way scatter plots of the standardized residuals and squared standardized residuals. Both suggested heteroscedasticity, but few potential outliers were identified based on standardized residuals > |3.5|, as decided a priori. Leverage and Cook’s D values suggested potential outliers exerted minimal influence (Cook’s D < 0.6), so all outliers were retained. Visual assessments of quantile versus quantile (Q-Q) plots and histograms of standardized residuals suggested violation of the normality assumption, and this was confirmed by significant Kolmogorov-Smirnov and Shapiro-Wilk tests (*P* < 0.001 for both). Comparing fully adjusted models with and without robust standard errors (SE) revealed no differences in conclusions based on coefficients with *P* < 0.05. However, to address heteroscedasticity, models with robust SE were selected and are reported in the accompanying manuscript.

Sensitivity analyses

We conducted sensitivity analyses using the same multiple linear regression models described in the main manuscript’s Methods section, but with PDI, hPDI, and uPDI scores based on absolute intakes while separately adjusting for energy intake in the models. Results showed nearly identical conclusions to the original models (refer to Table 4) across all models for all three indexes (see Supplemental Table S6). We compared quintile assignments for the two methods (absolute and energy-adjusted scoring) using Cohen’s kappa coefficients (κ), and results indicated fair to moderate agreement (PDI, κ = 0.48; hPDI, κ = 0.36; uPDI, κ = 0.35). Quintile distributions revealed most participant assignments that changed were shifted only one quintile in either direction.

**Supplemental Tables**

Table S1. Pearson’s *r* correlations for linked components from 2015-2016 USDA Food Patterns Equivalents Database (FPED1516) and the National Survey of Health and Development (NSHD)

| **FPED1516** | | | **NSHD** |  |
| --- | --- | --- | --- | --- |
| **Component** | **Description** | **Unit** | **Component (readily available or derived), g** | ***r*** |
| F_CITMLB | Intact fruits (whole or cut) of citrus, melons, and berries | cup eq | None |  |
| F_OTHER | Intact fruits (whole or cut); excluding citrus, melons, and berries | cup eq | None |  |
| F_JUICE | Fruit juices, citrus and non-citrus | cup eq | Fruit juice | 0.82 |
| F_TOTAL | Total intact or cut fruits and fruit juices | cup eq | Sum of fruit, dried fruit, fruit juice | 0.90 |
| V_DRKGR | Dark green vegetables | cup eq | None |  |
| V_REDOR_TOMATO | Tomatoes and tomato products | cup eq | Sum of tomatoes, tomato puree | 0.92 |
| V_REDOR_OTHER | Other red and orange vegetables, excluding tomatoes and tomato products | cup eq | Yellow/red/green vegetables | 0.90 |
| V_REDOR_TOTAL | Total red and orange vegetables (tomatoes + other red and orange) | cup eq | Sum of tomatoes, tomato puree, yellow/red/green vegetables | 0.91 |
| V_STARCHY_POTATO | White potatoes | cup eq | Total grams minus other components from “Potato” food groups | 0.98 |
| V_STARCHY_OTHER | Other starchy vegetables, excluding white potatoes | cup eq | None |  |
| V_STARCHY_TOTAL | Total starchy vegetables (white potatoes + other starchy) | cup eq | Total grams minus other components from “Potato” food groups | 0.96 |
| V_OTHER | Other vegetables not in the vegetable components listed above | cup eq | Sum of other vegetables, Brassicaceae | 0.77 |
| V_TOTAL | Total dark green, red and orange, starchy, and other vegetables; excludes legumes | cup eq | Sum of tomatoes, tomato puree, Brassicaceae, yellow/red/green vegetables, other vegetables, derived potatoes (see above) | 0.95 |
| V_LEGUMES | Legumes computed as vegetables | cup eq | Beans | 0.85 |
| G_WHOLE | Whole grains | oz eq | Whole grain | 0.87 |
| G_REFINED | Refined or non-whole grains | oz eq | Total grams minus other components from “Breads,” “Cereals,” “Cereal based,” “Biscuits,” and “Pastries” food groups | 0.84 |
| G_TOTAL | Total whole and refined grains | oz eq | Sum of whole grain, derived refined grain (see above) | 0.87 |
| PF_MEAT | Beef, veal, pork, lamb, game meat; excludes organ meats and cured meat | oz eq | Sum of beef, lamb, pork, other red meat | 0.93 |
| PF_CUREDMEAT | Cured/luncheon meat made from beef, pork, or poultry | oz eq | Sum of processed red meat, processed poultry, burgers, sausages | 0.88 |
| PF_ORGAN | Organ meat from beef, veal, pork, lamb, game, and poultry | oz eq | Offal | 0.89 |
| PF_POULT | Chicken, turkey, Cornish hens, and game birds; excludes organ meats and cured meat | oz eq | Sum of poultry, game birds | 0.95 |
| PF_SEAFD_HI | Seafood (finfish, shellfish and other seafood) high in n-3 fatty acids | oz eq | Sum of oily fish, canned tuna | 0.94 |
| PF_SEAFD_LOW | Seafood (finfish, shellfish and other seafood) low in n-3 fatty acids | oz eq | Sum of white fish, shellfish | 0.95 |
| PF_MPS_TOTAL | Total meat, poultry, seafood, organ meats, and cured meat | oz eq | Sum of beef, lamb, pork, processed red meat, other red meat, burgers, sausages, offal, poultry, processed poultry, game birds, white fish, oily fish, canned tuna, shellfish | 0.97 |
| PF_EGGS | Eggs (chicken, duck, goose, quail) and egg substitutes | oz eq | Total grams minus other components for “Egg” food group | 0.91 |
| PF_SOY | Soy products, excluding calcium fortified soy milk and immature soybeans | oz eq | None |  |
| PF_NUTSDS | Peanuts, tree nuts, and seeds, excludes coconut | oz eq | Nuts | 0.96 |
| PF_LEGUMES | Legumes computed as protein foods | oz eq | Beans | 0.85 |
| PF_TOTAL | Total meat, poultry, seafood, organ meats, cured meat, eggs, soy, and nuts and seeds; excludes legumes | oz eq | None |  |
| D_MILK | Fluid milk and calcium fortified soy milk | cup eq | Total grams minus other components for “Milk” food groups | 0.90 |
| D_YOGURT | Yogurt | cup eq | Total grams minus other components for “Yoghurt” food groups | 0.99 |
| D_CHEESE | Cheese | cup eq | Sum of cottage cheese, cheddar cheese, other cheese | 0.88 |
| D_TOTAL | Total milk, yogurt, cheese, and whey | cup eq | Sum of milk, yogurt, derived cheese (see above) plus total grams minus other components for “Milk based,” “Cream & fromage frais,” and “Ice cream & dairy desserts” food groups | 0.74 |
| OILS | Oils | grams | None |  |
| SOLID_FATS | Solid fats | grams | Saturated fatty acids | 0.78 |
| ADD_SUGARS | Foods defined as added sugars | tsp eq | Added sugars revised^1^ | 0.78 |
| A_DRINKS | Alcoholic beverages | # of drinks | Total grams minus other components for "Alcohol" food group | 0.93 |

eq, equivalent; oz, ounce; tsp, teaspoon

^1^ For items from the "Sugars - Pure sugars" and "Sugars - Other, incl. syrups, honey" food groups with added sugars = 0 g, values were corrected using NSHD data from previous years or, if none, conversions from FPED1516.

Table S2. Baseline comparisons (categorical) for those excluded from or included in the analytical sample^1^

|  | **Full cohort** |  | **Excluded** |  | **Included** |  |  |
| --- | --- | --- | --- | --- | --- | --- | --- |
| **Category, *n* (%)** | *n* = 5,360 |  | *n* = 2,846 |  | *n* = 2,514 | **χ^2^** | **V** |
| **Sex** |  |  |  |  |  | 34.94** | 0.08 |
| Male | 2,814 (52.5) |  | 1,602 (56.29) |  | 1,212 (48.21) |  |  |
| Female | 2,546 (47.5) |  | 1,244 (43.71) |  | 1,302 (51.79) |  |  |
| **Region of birth** |  |  |  |  |  | 21.83* | 0.06 |
| Wales | 287 (5.35) |  | 150 (5.27) |  | 137 (5.45) |  |  |
| Northern | 390 (7.28) |  | 193 (6.78) |  | 197 (7.84) |  |  |
| Yorkshire & Humberside | 449 (8.38) |  | 232 (8.15) |  | 217 (8.63) |  |  |
| Northwest England | 556 (10.37) |  | 297 (10.44) |  | 259 (10.30) |  |  |
| East Midlands | 342 (6.38) |  | 159 (5.59) |  | 183 (7.28) |  |  |
| West Midlands | 471 (8.79) |  | 268 (9.42) |  | 203 (8.07) |  |  |
| East Anglia | 191 (3.56) |  | 102 (3.58) |  | 89 (3.54) |  |  |
| Southeast England | 1712 (31.94) |  | 913 (32.08) |  | 799 (31.78) |  |  |
| Southwest England | 306 (5.71) |  | 148 (5.20) |  | 158 (6.28) |  |  |
| Scotland | 656 (12.24) |  | 384 (13.49) |  | 272 (10.82) |  |  |
| **Household social class, 11 y** |  |  |  |  |  |  |  |
| Total, *n* | 4,671 |  | 2,157 |  | 2,514 | 22.70** | 0.07 |
| Professional etc. | 289 (6.19) |  | 117 (5.42) |  | 172 (6.84) |  |  |
| Intermediate | 907 (19.42) |  | 385 (17.85) |  | 522 (20.76) |  |  |
| Skilled (Non-Manual) | 729 (15.61) |  | 311 (14.42) |  | 418 (16.63) |  |  |
| Skilled (Manual) | 1,554 (33.27) |  | 757 (35.10) |  | 797 (31.70) |  |  |
| Partly skilled | 881 (18.86) |  | 424 (19.66) |  | 457 (18.18) |  |  |
| Unskilled | 311 (6.66) |  | 163 (7.56) |  | 148 (5.89) |  |  |
| **Highest education attempted or achieved by 26 y** |  |  |  |  |  |  |  |
| Total, *n* | 4,562 |  | 2,089 |  | 2,473 | 84.03** | 0.14 |
| No qualifications or none attempted | 1,702 (37.31) |  | 916 (43.85) |  | 786 (31.78) |  |  |
| Vocational only | 144 (3.16) |  | 71 (3.40) |  | 73 (2.95) |  |  |
| Sub GCE or sub Burnham C | 208 (4.56) |  | 96 (4.60) |  | 112 (4.53) |  |  |
| O level or equivalent | 936 (20.52) |  | 399 (19.10) |  | 537 (21.71) |  |  |
| A level or equivalent | 674 (14.77) |  | 272 (13.02) |  | 402 (16.26) |  |  |
| Burnham A2 | 400 (8.77) |  | 144 (6.89) |  | 256 (10.35) |  |  |
| 1st Degree or graduate equivalent | 415 (9.10) |  | 158 (7.56) |  | 257 (10.39) |  |  |
| Higher degree, Masters | 38 (0.83) |  | 17 (0.81) |  | 21 (0.85) |  |  |
| Higher degree, doctorate | 45 (0.99) |  | 16 (0.77) |  | 29 (1.17) |  |  |

A level, Advanced level; GCE, General Certificate of Education; O level, Ordinary level; y, year

**^1^** Results from Pearson’s chi-square tests are presented (**P* < 0.01, ***P* < 0.001) along with Cramér's V effect sizes.

Table S3. Baseline comparisons (continuous) for those excluded from or included in the analytical sample

|  | **Full cohort** | |  | **Excluded** | |  | **Included** | |  |  |  |  |
| --- | --- | --- | --- | --- | --- | --- | --- | --- | --- | --- | --- | --- |
|  | *n* = 5,360 | |  | *n* = 2,846 | |  | *n* = 2,514 | |  | **Excluded vs. included^1^** | | |
|  | ***n*** | **Mean (SD)** |  | ***n*** | **Mean (SD)** |  | ***n*** | **Mean (SD)** |  | ***t*** | ***P*** | ***d*** |
| **Dietary index total scores at 4 y** |  |  |  |  |  |  |  |  |  |  |  |  |
| HEI-2020 (0-100 possible) | 4,597 | 45.89 (7.50) |  | 2,141 | 45.59 (7.42) |  | 2,456 | 46.16 (7.56) |  | -2.60 | 0.01 | -0.08 |
| PDI (18-90 possible) | 4,597 | 49.36 (7.02) |  | 2,141 | 49.61 (7.13) |  | 2,456 | 49.15 (6.92) |  | 2.20 | 0.03 | 0.07 |
| hPDI (18-90 possible) | 4,597 | 54.91 (5.21) |  | 2,141 | 55.03 (5.18) |  | 2,456 | 54.80 (5.23) |  | 1.54 | 0.12 | 0.05 |
| uPDI (18-90 possible) | 4,597 | 59.81 (5.89) |  | 2,141 | 59.82 (5.98) |  | 2,456 | 59.79 (5.82) |  | 0.17 | 0.86 | 0.01 |
| **Cognitive ability tests at 8 y** |  |  |  |  |  |  |  |  |  |  |  |  |
| Picture intelligence (0-60 possible) | 4,264 | 40.19 (9.48) |  | 1,929 | 38.91 (10.14) |  | 2,335 | 41.25 (8.77) |  | -7.96 | < 0.001 | -0.25 |
| Reading comprehension (0-35 possible) | 4,258 | 14.20 (7.78) |  | 1,929 | 13.27 (8.01) |  | 2,329 | 14.98 (7.50) |  | -7.16 | < 0.001 | -0.20 |
| Word reading (0-50 possible) | 4,258 | 17.02 (10.27) |  | 1,929 | 15.90 (10.51) |  | 2,329 | 17.94 (9.98) |  | -6.44 | < 0.001 | -0.22 |
| Vocabulary (0-50 possible) | 4,258 | 16.27 (5.99) |  | 1,929 | 15.75 (6.10) |  | 2,329 | 16.71 (5.87) |  | -5.22 | < 0.001 | -0.16 |

HEI, Healthy Eating Index; hPDI, Healthful Plant-based Diet Index; PDI, Plant-based Diet Index; uPDI, Unhealthful Plant-based Diet Index; y, year

**^1^** Results from independent means t-tests with unequal variances assumed are presented along with Cohen’s *d_s_* effect sizes.

Table S4. Dietary data contributed by participants in the analytical sample and the number of unique foods reported in the full cohort

|  |  |  |  | **Total days** |  | **Weekdays** |  | **Weekend days** |  | **Energy (kcal)** |  | **Unique foods reported in the full cohort (*n*)** | |
| --- | --- | --- | --- | --- | --- | --- | --- | --- | --- | --- | --- | --- | --- |
| **Age, y** | *n* | % |  | Mean (SD) |  | Mean (SD) |  | Mean (SD) |  | Mean (SD) |  | Recalls | Diaries |
| 4 | 2,456 | 97.69 |  | 1 (0.00) |  | 0.96 (0.18) |  | 0.04 (0.18) |  | 1,448.18 (341.50) |  | 449 | - |
| 36 | 1,860 | 73.99 |  | 6.66 (0.77) |  | 4.78 (0.58) |  | 1.88 (0.41) |  | 2,026.38 (614.94) |  | 966 | 1,082 |
| 43 | 2,420 | 96.26 |  | 5.63 (2.16) |  | 4.06 (1.59) |  | 1.57 (0.78) |  | 2,067.12 (379.52) |  | 984 | 1,008 |
| 53 | 1,569 | 62.41 |  | 4.98 (0.21) |  | 3.53 (0.73) |  | 1.45 (0.73) |  | 1,980.44 (508.34) |  | - | 1,645 |

y, year

Table S5. Results from adjusted multiple linear regression^1^ Model 5 with National Adult Reading Test scores as the dependent variable and different dietary indexes as primary predictors (*n* = 2,514)

|  |  | **HEI-2020** |  | **PDI** |  | **hPDI** |  | **uPDI** |
| --- | --- | --- | --- | --- | --- | --- | --- | --- |
| **Covariate groups & covariates** |  | *β* (95% CI) |  | *β* (95% CI) |  | *β* (95% CI) |  | *β* (95% CI) |
| **TYPICAL DIET** |  |  |  |  |  |  |  |  |
| **Diet index quintiles** (ref = Q1) |  |  |  |  |  |  |  |  |
| Q2 |  | 0.74 (-0.25, 1.74) |  | 0.24 (-0.69, 1.17) |  | -0.26 (-1.20, 0.68) |  | 0.06 (-0.81, 0.92) |
| Q3 |  | 1.14 (0.12, 2.17) |  | 0.61 (-0.33, 1.55) |  | -0.08 (-1.03, 0.87) |  | -0.19 (-1.08, 0.70) |
| Q4 |  | 1.90 (0.86, 2.93) |  | 0.38 (-0.55, 1.32) |  | 0.50 (-0.41, 1.41) |  | -1.24 (-2.21, -0.27) |
| Q5 |  | 2.25 (1.19, 3.30) |  | 1.17 (0.19, 2.15) |  | 1.42 (0.51, 2.33) |  | -1.55 (-2.55, -0.54) |
| **CHILDHOOD SOCIODEMOGRAPHICS** |  |  |  |  |  |  |  |  |
| **Sex** (ref = Male) |  |  |  |  |  |  |  |  |
| Female |  | 0.88 (0.05, 1.72) |  | 1.28 (0.46, 2.10) |  | 1.15 (0.32, 1.98) |  | 0.95 (0.12, 1.79) |
| **Region of birth** (ref = Wales) |  |  |  |  |  |  |  |  |
| Northern |  | 0.52 (-1.11, 2.16) |  | 0.44 (-1.20, 2.08) |  | 0.50 (-1.14, 2.14) |  | 0.61 (-1.03, 2.25) |
| Yorkshire and Humberside |  | 1.97 (0.46, 3.47) |  | 2.05 (0.55, 3.56) |  | 2.04 (0.53, 3.54) |  | 2.01 (0.51, 3.52) |
| Northwest England |  | -0.45 (-1.92, 1.03) |  | -0.40 (-1.86, 1.07) |  | -0.35 (-1.82, 1.13) |  | -0.39 (-1.87, 1.08) |
| East Midlands |  | 0.24 (-1.35, 1.83) |  | 0.31 (-1.28, 1.89) |  | 0.29 (-1.29, 1.87) |  | 0.31 (-1.26, 1.89) |
| West Midlands |  | -0.83 (-2.44, 0.77) |  | -0.81 (-2.42, 0.79) |  | -0.85 (-2.45, 0.75) |  | -0.78 (-2.38, 0.82) |
| East Anglia |  | -0.04 (-2.13, 2.05) |  | 0.06 (-2.06, 2.17) |  | 0.06 (-2.05, 2.16) |  | 0.04 (-2.07, 2.15) |
| South East England |  | -0.15 (-1.42, 1.12) |  | -0.05 (-1.32, 1.22) |  | -0.03 (-1.30, 1.24) |  | -0.05 (-1.33, 1.22) |
| South West England |  | 0.16 (-1.55, 1.86) |  | 0.25 (-1.45, 1.96) |  | 0.27 (-1.45, 1.98) |  | 0.16 (-1.55, 1.86) |
| Scotland |  | -0.13 (-1.56, 1.30) |  | -0.13 (-1.56, 1.30) |  | -0.11 (-1.55, 1.32) |  | -0.20 (-1.64, 1.24) |
| **Household social class, 11 y** (ref = Professional, etc.) |  |  |  |  |  |  |  |  |
| Intermediate |  | -0.05 (-1.20, 1.10) |  | -0.10 (-1.26, 1.05) |  | -0.16 (-1.30, 0.98) |  | -0.14 (-1.27, 1.00) |
| Skilled (non-manual) |  | -0.56 (-1.78, 0.65) |  | -0.71 (-1.94, 0.52) |  | -0.72 (-1.94, 0.49) |  | -0.58 (-1.78, 0.63) |
| Skilled (manual) |  | -2.20 (-3.39, -1.01) |  | -2.42 (-3.61, -1.22) |  | -2.39 (-3.57, -1.21) |  | -2.29 (-3.48, -1.11) |
| Partly skilled |  | -2.17 (-3.46, -0.89) |  | -2.38 (-3.67, -1.08) |  | -2.33 (-3.61, -1.04) |  | -2.21 (-3.49, -0.92) |
| Unskilled |  | -3.65 (-5.34, -1.95) |  | -3.94 (-5.66, -2.23) |  | -3.94 (-5.64, -2.24) |  | -3.81 (-5.51, -2.11) |
| **ADULTHOOD SOCIODEMOGRAPHICS** |  |  |  |  |  |  |  |  |
| **Highest education level attempted or achieved by 43 y** (ref = No qualifications or none attempted) |  |  |  |  |  |  |  |  |
| Vocational only |  | 1.51 (0.23, 2.79) |  | 1.54 (0.26, 2.83) |  | 1.52 (0.24, 2.81) |  | 1.51 (0.23, 2.79) |
| Sub GCE or sub Burnham C |  | 4.99 (3.45, 6.52) |  | 5.07 (3.53, 6.61) |  | 5.01 (3.48, 6.55) |  | 5.03 (3.49, 6.58) |
| O level or equivalent |  | 5.15 (4.15, 6.15) |  | 5.28 (4.28, 6.28) |  | 5.30 (4.30, 6.30) |  | 5.22 (4.22, 6.23) |
| A level or equivalent |  | 7.05 (6.00, 8.09) |  | 7.16 (6.10, 8.21) |  | 7.14 (6.09, 8.19) |  | 7.10 (6.05, 8.14) |
| Burnham A2 |  | 7.55 (6.29, 8.81) |  | 7.65 (6.38, 8.92) |  | 7.71 (6.45, 8.97) |  | 7.68 (6.40, 8.96) |
| 1st Degree or graduate equivalent |  | 10.25 (9.03, 11.46) |  | 10.50 (9.29, 11.72) |  | 10.45 (9.24, 11.66) |  | 10.40 (9.18, 11.62) |
| Higher degree, Masters or doctorate |  | 10.67 (8.47, 12.87) |  | 11.03 (8.82, 13.24) |  | 10.96 (8.76, 13.17) |  | 10.89 (8.68, 13.10) |
| **Highest social class (head of household) by 53 y** (ref = Professional, etc.) |  |  |  |  |  |  |  |  |
| Intermediate |  | -0.58 (-1.30, 0.15) |  | -0.63 (-1.36, 0.09) |  | -0.64 (-1.36, 0.08) |  | -0.65 (-1.37, 0.08) |
| Skilled (non-manual) |  | -1.68 (-2.82, -0.54) |  | -1.87 (-3.02, -0.72) |  | -1.78 (-2.92, -0.65) |  | -1.84 (-2.98, -0.69) |
| Skilled (manual) |  | -4.34 (-5.46, -3.22) |  | -4.61 (-5.73, -3.49) |  | -4.56 (-5.67, -3.44) |  | -4.43 (-5.55, -3.32) |
| Partly skilled or unskilled |  | -4.95 (-7.77, -2.13) |  | -5.08 (-7.91, -2.26) |  | -5.14 (-7.97, -2.31) |  | -5.01 (-7.82, -2.20) |
| **LIFESTYLE** |  |  |  |  |  |  |  |  |
| **Cigarette status, 53 y** (ref = Current smoker) |  |  |  |  |  |  |  |  |
| Ex-smoker |  | 0.54 (-0.32, 1.41) |  | 0.73 (-0.14, 1.60) |  | 0.75 (-0.11, 1.61) |  | 0.77 (-0.09, 1.63) |
| Never smoked |  | 0.35 (-0.50, 1.19) |  | 0.56 (-0.29, 1.41) |  | 0.64 (-0.21, 1.48) |  | 0.67 (-0.18, 1.51) |
| **Alcohol, quintiles of cumulative mean intake, 36-53 y** (ref = Q1) |  |  |  |  |  |  |  |  |
| Q2 |  | 0.73 (-0.24, 1.70) |  | 0.80 (-0.18, 1.77) |  | 0.87 (-0.10, 1.83) |  | 0.72 (-0.26, 1.69) |
| Q3 |  | 1.84 (0.88, 2.79) |  | 2.02 (1.06, 2.98) |  | 2.03 (1.08, 2.99) |  | 1.91 (0.95, 2.86) |
| Q4 |  | 1.75 (0.77, 2.73) |  | 1.93 (0.94, 2.92) |  | 1.96 (0.97, 2.95) |  | 1.82 (0.84, 2.80) |
| Q5 |  | 2.14 (1.04, 3.23) |  | 2.33 (1.21, 3.44) |  | 2.31 (1.20, 3.42) |  | 2.30 (1.19, 3.41) |
| **Physical activity status, 53 y** (ref = Inactive) |  |  |  |  |  |  |  |  |
| Less active (1-4 times) |  | 1.55 (0.71, 2.39) |  | 1.64 (0.80, 2.47) |  | 1.64 (0.80, 2.48) |  | 1.65 (0.81, 2.49) |
| Most active (5+ times) |  | 0.74 (0.05, 1.42) |  | 0.90 (0.21, 1.58) |  | 0.83 (0.15, 1.51) |  | 0.88 (0.20, 1.56) |
| **LEISURE ACTIVITIES** |  |  |  |  |  |  |  |  |
| **Intellectual:** musical/artistic/ creative or constructive, 53 y (ref = None) |  |  |  |  |  |  |  |  |
| One activity type |  | 1.41 (0.70, 2.13) |  | 1.41 (0.69, 2.12) |  | 1.37 (0.66, 2.08) |  | 1.37 (0.66, 2.09) |
| Both activity types |  | 1.10 (0.35, 1.85) |  | 1.19 (0.45, 1.94) |  | 1.08 (0.33, 1.83) |  | 1.10 (0.35, 1.86) |
| **Social:** Friends/relatives seen at least monthly, 53 y (ref = None) |  |  |  |  |  |  |  |  |
| One or more |  | -0.77 (-2.56, 1.02) |  | -0.81 (-2.59, 0.97) |  | -0.81 (-2.61, 0.99) |  | -0.79 (-2.56, 0.99) |
| **HEALTH STATUS (53 y)** |  |  |  |  |  |  |  |  |
| BMI (continuous) |  | 0.03 (-0.04, 0.09) |  | 0.03 (-0.03, 0.10) |  | 0.03 (-0.03, 0.10) |  | 0.02 (-0.05, 0.08) |
| Diabetes (ref = No) |  | 0.50 (-1.28, 2.28) |  | 0.63 (-1.13, 2.39) |  | 0.62 (-1.15, 2.39) |  | 0.46 (-1.32, 2.23) |
| Headache/migraine (ref = No) |  | 0.63 (-0.09, 1.34) |  | 0.55 (-0.17, 1.27) |  | 0.58 (-0.13, 1.29) |  | 0.65 (-0.07, 1.36) |
| Heart attack (ref = No) |  | -0.84 (-3.07, 1.39) |  | -0.78 (-3.03, 1.48) |  | -0.81 (-3.04, 1.42) |  | -0.75 (-2.96, 1.46) |
| High BP (ref = No) |  | -1.01 (-1.77, -0.25) |  | -1.06 (-1.83, -0.30) |  | -1.02 (-1.78, -0.26) |  | -1.06 (-1.82, -0.30) |
| Epilepsy medication (ref = No) |  | -2.87 (-6.39, 0.65) |  | -2.82 (-6.32, 0.67) |  | -2.78 (-6.20, 0.65) |  | -2.75 (-6.21, 0.71) |
| Nervous/emotional trouble or anxiety/depression (ref = No) |  | 0.00 (-0.69, 0.69) |  | 0.05 (-0.64, 0.74) |  | 0.01 (-0.68, 0.70) |  | 0.04 (-0.65, 0.73) |
| Stroke (ref = No) |  | 0.38 (-2.53, 3.28) |  | 0.46 (-2.50, 3.41) |  | 0.25 (-2.70, 3.19) |  | 0.54 (-2.44, 3.51) |

A level, Advanced level; BP, blood pressure; d, day; GCE, General Certificate of Education; HEI, Healthy Eating Index; hPDI, Healthful Plant-based Diet Index; O level, Ordinary level; PDI, Plant-based Diet Index; ref, reference group; uPDI, Unhealthful Plant-based Diet Index; y, years

^1^ Adjusted mean difference beta coefficients and 95% CIs are presented from linear regression models with robust standard errors.

Table S6. Select results from sensitivity analyses using plant-based dietary index scores based on absolute dietary intakes: multiple linear regression models^1^ with National Adult Reading Test scores as the dependent variable, dietary index quintiles as the primary independent variables, and additional covariate groups^2^ added to each model (*n* = 2,514)

|  | **Model 1**  ***Energy*** | **Model 2**  ***1 + Sociodem.*** | **Model 3**  ***2 + Lifestyle*** | **Model 4**  ***3 + Leisure activities*** | **Model 5**  ***4 + Health status*** | **Model 6**  ***5 + Childhood cog.*** |
| --- | --- | --- | --- | --- | --- | --- |
| **Dietary index** | *ꞵ* (95% CI) | *ꞵ* (95% CI) | *ꞵ* (95% CI) | *ꞵ* (95% CI) | *ꞵ* (95% CI) | *ꞵ* (95% CI) |
| **PDI, *R*^2^** | **0.0249** | **0.3788** | **0.3916** | **0.3966** | **0.4001** | **0.5170** |
| Q2 | 1.56 (0.37, 2.75) | 0.58 (-0.36, 1.53) | 0.44 (-0.51, 1.39) | 0.41 (-0.54, 1.36) | 0.37 (-0.58, 1.32) | 0.55 (-0.30, 1.39) |
| Q3 | 2.23 (1.10, 3.36) | 0.73 (-0.18, 1.64) | 0.55 (-0.37, 1.47) | 0.48 (-0.44, 1.40) | 0.49 (-0.43, 1.41) | 0.44 (-0.37, 1.26) |
| Q4 | 2.64 (1.39, 3.89) | 1.01 (-0.01, 2.02) | 0.72 (-0.30, 1.75) | 0.69 (-0.33, 1.70) | 0.63 (-0.39, 1.65) | 0.47 (-0.44, 1.38) |
| Q5 | 4.58 (3.36, 5.80) | 1.52 (0.50, 2.53) | 1.31 (0.27, 2.35) | 1.22 (0.18, 2.25) | 1.17 (0.13, 2.21) | 0.79 (-0.12, 1.70) |
| **hPDI, *R*^2^** | **0.0273** | **0.3792** | **0.3921** | **0.3968** | **0.4002** | **0.5172** |
| Q2 | 0.71 (-0.48, 1.91) | 0.13 (-0.84, 1.11) | 0.09 (-0.87, 1.06) | 0.10 (-0.87, 1.07) | 0.07 (-0.90, 1.03) | -0.13 (-1.00, 0.73) |
| Q3 | 2.39 (1.15, 3.63) | 0.50 (-0.51, 1.50) | 0.37 (-0.65, 1.39) | 0.35 (-0.66, 1.37) | 0.29 (-0.73, 1.30) | 0.03 (-0.89, 0.94) |
| Q4 | 2.80 (1.54, 4.06) | 0.53 (-0.51, 1.57) | 0.40 (-0.64, 1.44) | 0.33 (-0.70, 1.36) | 0.27 (-0.76, 1.30) | 0.09 (-0.85, 1.04) |
| Q5 | 5.10 (3.76, 6.44) | 1.71 (0.60, 2.81) | 1.49 (0.37, 2.62) | 1.29 (0.17, 2.41) | 1.19 (0.07, 2.32) | 0.77 (-0.26, 1.80) |
| **uPDI, *R*^2^** | **0.0654** | **0.3834** | **0.3955** | **0.3999** | **0.4035** | **0.5186** |
| Q2 | -2.66 (-3.72, -1.61) | -0.68 (-1.57, 0.21) | -0.57 (-1.46, 0.32) | -0.47 (-1.36, 0.42) | -0.46 (-1.36, 0.44) | -0.06 (-0.89, 0.77) |
| Q3 | -3.71 (-4.81, -2.62) | -1.03 (-1.95, -0.11) | -0.88 (-1.80, 0.04) | -0.80 (-1.71, 0.12) | -0.77 (-1.69, 0.15) | -0.59 (-1.43, 0.24) |
| Q4 | -5.28 (-6.39, -4.18) | -1.80 (-2.75, -0.86) | -1.67 (-2.62, -0.72) | -1.50 (-2.45, -0.55) | -1.52 (-2.47, -0.57) | -0.91 (-1.78, -0.03) |
| Q5 | -7.27 (-8.44, -6.11) | -2.51 (-3.52, -1.49) | -2.23 (-3.25, -1.20) | -2.06 (-3.09, -1.03) | -2.03 (-3.06, -1.00) | -1.32 (-2.25, -0.39) |

hPDI, Healthful Plant-based Diet Index; PDI, Plant-based Diet Index; Q, quintile; uPDI, Unhealthful Plant-based Diet Index

^1^ Mean differences (beta coefficients), 95% CIs, and *R*^2^ results are presented from linear regression models with robust standard errors and quintile 1 as the reference group.

^2^ Covariates for each model are as follows. **Model 1**: Crude model (dependent variable = NART scores; independent variables = dietary index quintiles based on absolute dietary intakes) plus adjustment for cumulative mean energy (kcal); **Model 2**: Model 1 plus all sociodemographic factors (Childhood: sex, region of birth, social class; Adulthood: highest education level, highest household social class); **Model 3**: Model 2 plus lifestyle factors (cigarette status, alcohol cumulative mean quintiles, exercise status); **Model 4**: Model 3 plus leisure activity factors (intellectual and social activities); **Model 5**: Model 4 plus health status measures (BMI, diabetes, headache/migraine, heart attack, high blood pressure, epilepsy medication use, nervous condition, stroke); **Model 6**: Model 5 plus childhood cognitive ability.

**Supplemental Figures**

Figure S1. Health Eating Index (HEI)-2020 total scores over time. Unadjusted medians and IQRs are presented for quintile groups (Q; based on cumulative mean HEI scores).

Figure S2. Overall Plant-based Diet Index (PDI) total scores over time. Unadjusted medians and IQRs are presented for quintile groups (Q; based on cumulative mean PDI scores).

Figure S3. Healthful Plant-based Diet Index (hPDI) total scores over time. Unadjusted medians and IQRs are presented for quintile groups (Q; based on cumulative mean hPDI scores).

Figure S4. Unhealthful Plant-based Diet Index (uPDI) total scores over time. Unadjusted medians and IQRs are presented for quintile groups (Q; based on cumulative mean uPDI scores).

Figure S5. Radar plot of Healthy Eating Index (HEI)-2020 component scores for each quintile compared to perfect scores. Results presented are unadjusted median scores from the analytical sample (*n* = 2,514); Higher scores represent closer alignment with guidelines: higher intakes of adequacy components (i.e., total vegetables clockwise to fatty acids) and lower intakes of moderation components (i.e., sodium clockwise to added sugar).

Figure S6. Radar plot of Plant-based Diet Index (PDI) component scores for each quintile compared to perfect scores. Results presented are unadjusted median scores from the analytical sample (*n* = 2,514); Higher scores for all plant-based food groups indicate higher intakes (i.e., whole grains clockwise to sweets & desserts), while higher scores for animal-based food groups indicate lower intakes (i.e., animal fat clockwise to misc. animal-based foods).

Figure S7. Radar plot of Healthful Plant-based Diet Index (hPDI) component scores for each quintile compared to perfect scores. Results presented are unadjusted median scores from the analytical sample (*n* = 2,514); Higher scores for all healthy plant-based food groups indicate higher intakes (i.e., whole grains clockwise to tea & coffee), while higher scores for less healthy plant-based food groups (i.e., fruit juices clockwise to sweets & desserts), and animal-based food groups (i.e., animal fat clockwise to misc. animal-based foods) indicate lower intakes.

Figure S8. Radar plot of Unhealthful Plant-based Diet Index (uPDI) component scores for each quintile compared to perfect scores. Results presented are unadjusted median scores from the analytical sample (*n* = 2,514); Higher scores for all less healthy plant-based food groups indicate higher intakes (i.e., fruit juices clockwise to sweets & desserts), while higher scores for healthy plant-based food groups (i.e., whole grains clockwise to tea & coffee) and animal-based food groups (i.e., animal fat clockwise to misc. animal-based foods) indicate lower intakes.
